# Tailoring Peptide Coacervates for Advanced Biotechnological Applications: Enhancing Control, Encapsulation, and Antioxidant Properties

**DOI:** 10.1021/acsami.5c02367

**Published:** 2025-04-28

**Authors:** Daniel Boas, Mohammad Taha, Edit Y. Tshuva, Meital Reches

**Affiliations:** † Institute of Chemistry, 26742 The Hebrew University of Jerusalem, Jerusalem 9190401, Israel; ‡ The Center for Nanoscience and Nanotechnology, The Hebrew University of Jerusalem, Jerusalem 9190401, Israel

**Keywords:** peptide coacervates, controlled deposition, dietary supplements, concentration gradient, antioxidant

## Abstract

The increasing interest in protein and peptide coacervates is accompanied by the development of various applications, from drug delivery to biosensor preparation. However, the impact of peptide end groups and charges on coacervation remains unclear. For this purpose, we designed four peptide derivatives with varying end groups and net charges. These inherently fluorescent peptides readily formed coacervates in solution or during evaporation. The ability to control the coacervation process, the coacervate’s appearance, and the encapsulation capabilities were thoroughly investigated. The coacervates displayed significant antioxidant properties, protecting the encapsulated material. Additionally, control of the deposition of coacervates on surfaces was achieved. These abilities highlight the potential of these coacervates in biotechnological applications, including biosensor development and the delivery of compounds such as drugs and dietary supplements. Exploiting the dynamic characteristics of coacervates with the unique properties of these peptides underscores their practical advantages.

## Graphical abstract

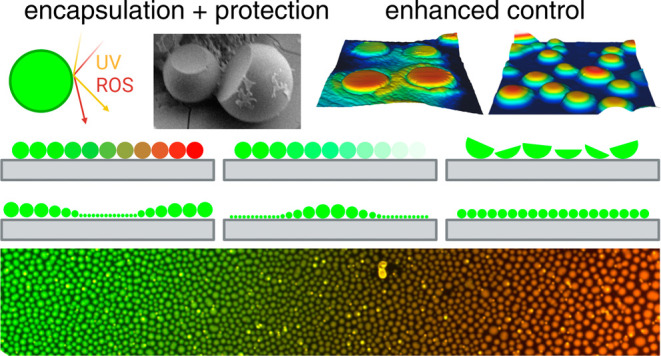

## Introduction

Liquid–liquid phase separation (LLPS) occurs when a homogeneous solution containing different solutes undergoes phase separation into at least two immiscible liquids.^1^ Coacervates form in a specific process of LLPS and are usually described as the dense phase rich in macromolecules such as proteins, polymers, or nucleic acids that condensate out of the dilute phase.^2^ LLPS is considered a significant process in biological systems, leading to the formation of various membrane-less organelles in the cells.^2^ Many regions and compartments in the cells exhibit liquid phase characteristics, such as the nucleolus and Cajal bodies in the nucleus and stress granules and P-bodies in the cytoplasm.^3,4^ LLPS is involved in different diseases including cancer, infectious diseases, and various neurodegenerative diseases.^5^ In several cases, a link was found between the formation of coacervates through LLPS, the following liquid-to-solid transition of these coacervates into aggregates, and neurodegenerative diseases. Such cases include the microtubule-associated protein Tau in Alzheimer’s disease, α-synuclein in Parkinson’s disease, and poly glutamine in Huntington’s disease.^6–9^

Coacervates can form in processes of complex or simple coacervation.^10^ In complex coacervation, two solutes condense together due to attractive interactions. An example of complex coacervates can be found in polymers with opposite charges such as poly aspartic acid or poly glutamic acid with poly arginine or poly lysine.^11^ In simple coacervation, only one solute is involved, and its attractive intermolecular interactions cause it to condense at certain conditions, i.e., solute concentration, pH, and ionic strength. Various interactions can contribute to the formation of coacervates depending on the solute, including ionic interactions, hydrogen bonds, π–π stacking, and cation–π interactions. The model of associative polymers can be used to describe the behavior of polymers and proteins in LLPS.^12–14^ This model depicts molecules with functional groups called stickers separated by spacers. The stickers form reversible bonds with each other that lead to intermolecular clusters and finally to phase separation. The hydrophobicity of the sticker and the polarity of the spacer were shown to influence the ability of the solute to form stable coacervates.^15^

For most biological processes, the investigation of LLPS occurs in solution. However, coacervates can form during evaporation of the solution, which increases the concentration of the solute and the ionic strength. Evaporation could have been crucial for prebiotic processes and since LLPS was shown to form membrane-less organelles inside cells, coacervates are considered as potential protocell models.^10,16^ During evaporation of a sessile droplet, preferential evaporation of the edge of the droplet causes capillary effects that replenish the liquid.^17,18^ This capillary flow transports the particles to the edges, forming a coffee stain effect. This effect can be suppressed by introducing surface tension gradients that generate inward Marangoni flows.^17^ These effects can be used to control the deposition of particles on surfaces, which is important for surface coating and surface patterning applications.^17^ The capillary and Marangoni flows can also affect the deposition of coacervates on surfaces.

Coacervates can be used for various applications, from catalysis to 3D bioprinting,^15,19–21^ biosensing applications,^22^ deposition of pesticides,^23^ and formation of underwater adhesives.^24,25^ They can also be used for different biomedical applications such as drug delivery, protein delivery, and drug protection.^26–29^ In delivery applications, coacervates can be advantageous because of their encapsulation ability. Additionally, they can be especially useful if they can protect the encapsulated material from degradation due to oxidation or exposure to UV light.

While extensive research has been conducted on coacervates, the effect of the charge of the molecules on their ability to condensate remains unclear. Peptide coacervates have been investigated, but the influence of the different possible end groups on the coacervation process has not been studied. Similarly, a comparison of the different coacervation processes in solution and during evaporation was not conducted. Our goal in this research was to study the impact of the charge of the peptides on the coacervation and to compare both coacervation processes. This information could not only expand our knowledge of this phenomenon but also lead to improved applications that these coacervates may provide.

In this research, we designed a controllable coacervate system of four peptide derivatives with a minimal sticker-and-spacer motif. The formed coacervates exhibited efficient encapsulation capabilities with strong antioxidant activity that can protect the encapsulated material. The formation and appearance of the coacervates can be controlled, as well as their deposition on surfaces. The coacervates can be adjusted in multiple ways by altering various parameters, such as the end groups, coacervation process, solution conditions, deposition process, and encapsulated material. This adjustability greatly enhances the potential of this system to be used for applications. For example, the delivery and release of drugs or dietary supplements can be controlled through the stability of the coacervates. Additionally, controlling the size and shape of the coacervates, as well as the concentration of the encapsulated material, can expand the design possibilities of sensors and improve their efficiency. This work utilizes the dynamic properties of coacervates, such as their ability to condense and encapsulate additional compounds with the thermodynamic stability of the resulting dried or aged particles.

## Results and Discussion

### Peptide Design

Four peptide derivatives were designed and investigated for their coacervation properties: NH_2_-Trp-(His)_2_-Trp-COOH (pep1), NH_2_-Trp-(His)_2_-Trp-CONH_2_ (pep2), Ac-Trp-(His)_2_-Trp-CONH_2_ (pep3), and Ac-Trp-(His)_2_-Trp-COOH (pep4) (Figure 1A). The first peptide (pep1) has an amine and a carboxylic group (which provide positive and negative charges at neutral pH, respectively). These groups are substituted with neutral end groups in the other derivatives, providing a collection of zwitterionic, cationic, anionic, and nonionic peptides. The peptides can be regarded as minimal sticker-and-spacer motifs, which are found in several proteins prone to coacervate.^15^ The tryptophan residues at the termini act as the hydrophobic stickers, while the histidines in the middle act as the hydrophilic spacer. The different combinations of end-groups alter the net charge of the peptides at different pH values (Figure 1B), providing a better grasp of the effects of charges on the ability of peptides to form simple coacervates. Furthermore, it allows for a comparison of the abilities of nonionic, cationic, anionic, and zwitterionic peptides to form these coacervates. A 3-(4,5-dimethylthiazol-2-yl)-2,5-diphenyltetrazolium bromide (MTT) assay showed that the four peptides did not display any cytotoxicity even at relatively high concentrations (Figure 1C), indicating their potential to be used for medicinal purposes.

**Figure 1:**
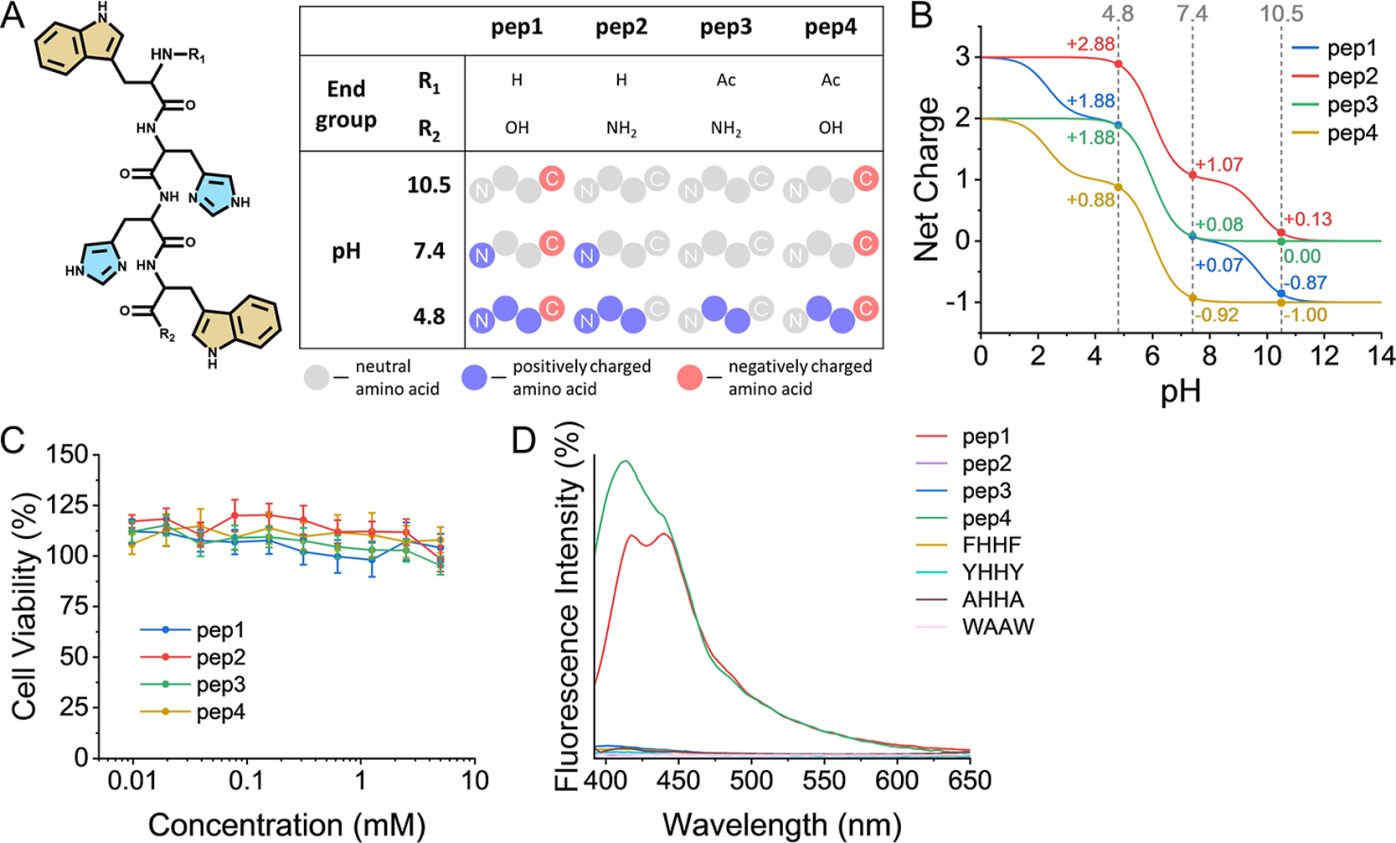
Four investigated peptide derivatives. (A) Common chemical structure shared by the four peptides (left) and a table describing the different end groups of the peptides and their differing charges at the three pH values investigated in this research (right). (B) Net charges of the four peptides versus the pH. (C) Cell viability results with human ovarian A2780 cells for different concentrations of each of the four peptides, following a three-day incubation period as analyzed by the MTT assay. The data points and error bars represent the arithmetic mean and standard error values of nine repeats. (D) Fluorescence intensity percentage of the four peptides and of the control peptides at pH 7.4.

From this group of peptide derivatives, pep1 and pep4 display strong inherent fluorescence (Figure 1D). The fluorescence emission appeared as two peaks, similar to cyan fluorescent proteins.^30^ In comparison, the peptide NH_2_-Trp-Ala-Ala-Trp-COOH (WAAW), without histidine residues, showed no fluorescence, while NH_2_-Ala-His-His-Ala-COOH (AHHA), without tryptophan moieties, showed only low fluorescence intensity, suggesting that both tryptophan and histidine side chains are necessary for the fluorescence (Figure S1A). Since pep1 and pep4 displayed the highest fluorescence intensity, it appears that the presence of the carboxyl group or of its negative charge is also necessary for the fluorescence. On the other hand, positive charges appear to diminish the fluorescence, as can be observed in the higher fluorescence intensity of pep4 when compared to pep1 (with net charges of −0.92 and +0.07, respectively). Similarly, a decrease in the pH value accompanied by an increase in the net charge of pep1, appears to reduce its fluorescence intensity (Figure S1B).

These observations may be explained by the electron-withdrawing characteristics of the carboxylate versus the electron-donating characteristics of the amide. The carboxylate may lower the energy of the excited state of the peptide via inductive effects, thus increasing the fluorescence of pep1 and pep4 in comparison to pep2 and pep3, by reducing nonradiative decay.^31^ Similarly, when pep1 and pep4 are compared, the acetyl group of pep4 is electron-withdrawing, while the amine of pep1 is electron-donating. Additionally, the carboxylate can form stronger hydrogen bonds, which may result in a more rigid structure with increased fluorescence.^32,33^

### Two Coacervation Processes

The coacervation was performed in two different processes: coacervation in solution and coacervation during evaporation. In solution a high ionic strength is usually required to promote the LLPS process as it screens the ionic interactions of the peptides, reducing their solubility, and allowing the stickers to employ aromatic and hydrophobic interactions for the peptides to approach each other and coacervate (Figure 2A). The formed coacervates coalesce, indicating that they are liquid (Figure 2B). Cryo-SEM images of the coacervates also show the coalescence of the droplets (Figures 2C and S2). Additionally, very small coacervates at the size range of ∼300 nm can be seen, which may indicate that the coacervation initiates with much smaller particles that coalesce until they reach the size of several microns observed in the microscope images.

**Figure 2:**
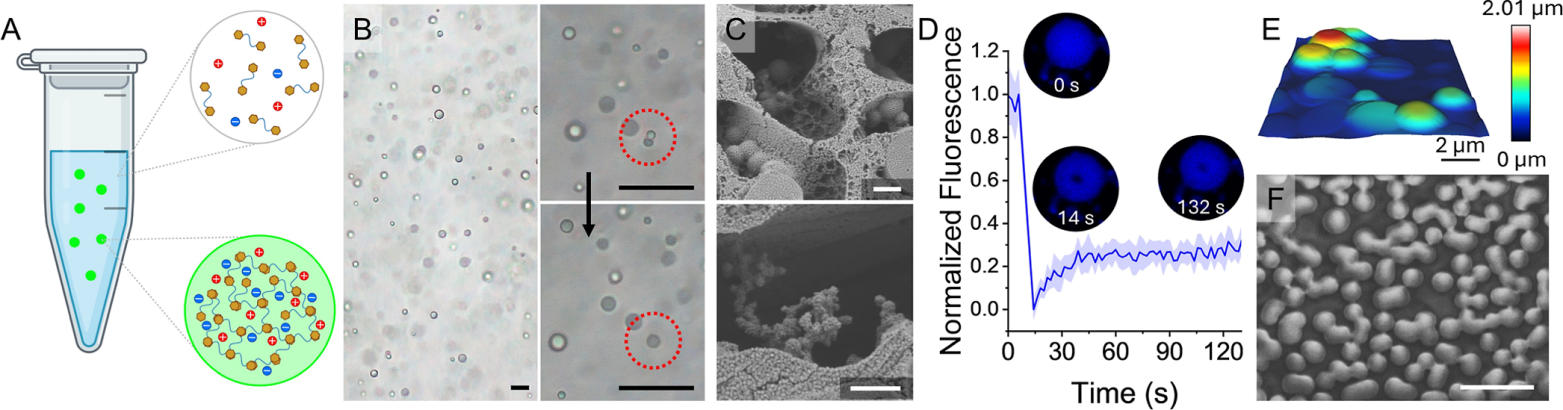
Coacervation in solution. (A) Scheme showing the formation of the sticker-and-spacer peptide coacervates in a solution with high ionic strength. (B) Transmitted light microscopy image of coacervates of pep4 (5 mM) in acetate buffer (pH 4.8, 25 mM, 3 M NaCl) with zoomed-in images showing coalescence of the coacervates (scale bar: 20 μm). (C) Cryo-SEM images of the coacervates of pep4 (scale bars: 1 μm). (D) FRAP measurements of coacervates of pep4. Insets: representative confocal microscopy images of a bleached coacervate at different time points. (E) AFM image of coacervates of pep4. (F) SEM image of coacervates of pep4 (scale bar: 10 μm).

Despite the ability of the coacervates to coalesce, they appear to partially solidify, while preserving their spherical shape. This can be seen by a fluorescence recovery after photobleaching (FRAP) measurements that show low fluorescence recovery of ∼30%, indicating that the coacervates are solidifying in the solution (Figure 2D). Upon drying, the coacervates do not disassemble but remain as spherical particles (Figure 2E,F). The solidification of the coacervates appeared to be accelerated at alkaline pH and at high ionic strength (Figure S15A). It is possible that the higher net charge of the peptides at acidic or neutral pH retains water molecules inside the coacervates, slowing the solidification process. Additionally, the high ionic strength may generate osmotic pressure that ejects the water from the coacervates, accelerating their solidification.

In the second process, a solution of the peptides is allowed to evaporate at room temperature. The evaporation causes an outward capillary flow that localizes a high concentration of the peptide and the salt ions at the perimeter of the droplet, which, in turn, forms coacervates (Figure 3A and Video S1). These coacervates coalesce in a fashion similar to that of those formed in solution (Figure 3B and Video S2).

**Figure 3:**
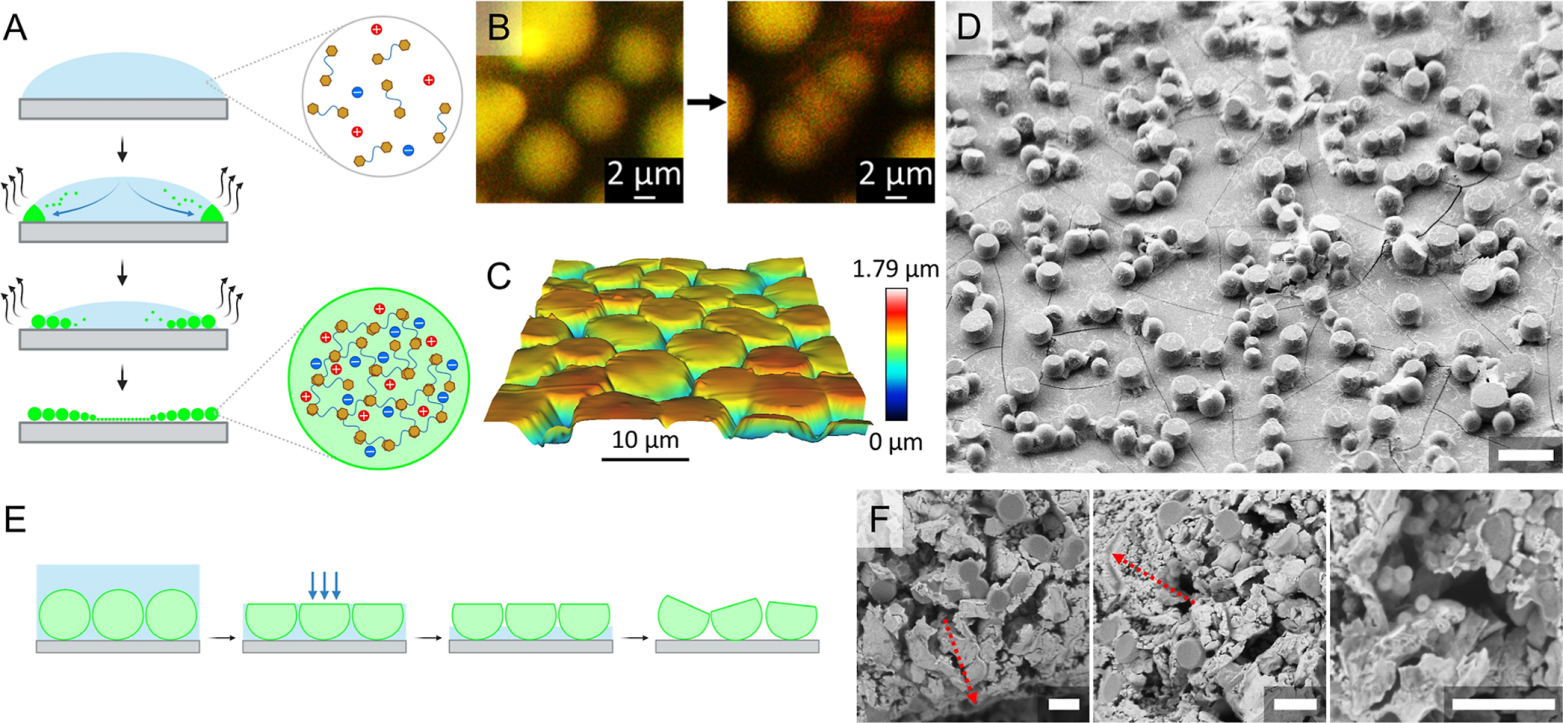
Coacervation during evaporation. (A) Scheme showing the formation of the sticker-and-spacer peptide coacervates in an evaporating droplet. (B) Fluorescence microscopy images showing three coacervates (left) that coalesce together (right). The coacervates were made with pep1 (8 mM) in phosphate buffer (pH 7.4, 25 mM, 0.4 M NaCl) with rhodamine 110 chloride (80 mg/L). (C) AFM image of coacervates of pep1. (D) SEM image of coacervates of pep1 (scale bars: 10 μm). (E) Scheme describing the process that forms the hemispherical structures. (F) Cryo-SEM images of the coacervates of pep4 (scale bar: 1 μm). Red arrows point to where the water–air interface was located.

The same solidification behavior can be observed for the coacervates that formed during evaporation. When the solution is placed on a hydrophobic substrate such as parafilm (contact angle 106°), which lowers the spread of the droplet, allowing the solution to age for a longer duration before complete evaporation, a higher intensity of the fluorescence of the peptide can be observed and the spherical shape of the coacervates is distorted (Figure S3A). This further implies that the coacervates solidify as they age, beginning from their outer surface. When mixed with a fluorescent dye, lines can be observed where two or more droplets coalesced (Figure S3B and Video S2). This also indicates the formation of a semisolid outer shell that allows for coalescence but does not fully behave like a liquid, similar to the FRAP results in Figure 2D.

A unique process causes the coacervates formed during the evaporation process to appear as hemispheres (Figure 3C,D). This phenomenon can be explained by the semisolid behavior of the coacervates. As the water level decreases during evaporation, the surface tension pushes the coacervates downward, forming a flat surface at the water–air interface (Figure 3E). The same can be observed in Figure S4 where smaller coacervates located underneath larger ones did not interact with the interface and remained spherical. To show that this is the correct process in which the hemispheres are formed and that they are not formed due to fracture of previously coalesced semisolid droplets, we performed Cryo-SEM imaging on a solution of coacervates formed during evaporation. The resulting images show that the flat surfaces of different hemispheres are pointing toward the water–air interface, in accordance with the slope of the solution droplet (Figures 3F and S5). Similar to the coacervates formed in solution, small coacervates were observed in the size range of ∼100 nm.

### Coacervation Phase Diagrams

Figure 4A shows the phase diagrams of the four peptides in solutions with different peptide concentrations and ionic strength values. The phase diagrams indicate that high pH values (10.5 > 7.4 > 4.8) promote coacervation, as coacervates form at low ionic strengths and even in buffer solutions with no added salt. The net charge of all four peptides decreases as the pH values increase. The lower charge of the peptides requires lower ionic strength to form coacervates as fewer ions are needed to screen the charges in order to condense the peptides. This behavior can be observed by comparing the coverage of the phase diagrams with the net charge values of the peptides (Figures 4C and S6). The coacervation capabilities (indicated by a higher coverage of the phase diagrams) are stronger when the peptides have net charges closer to zero. For example, the most favorable conditions for coacervation are found for pep2 at pH 10.5, in which it has a net charge of +0.13. On the other hand, at pH 4.8, pep2 has the highest net charge of all peptides, +2.88, and it cannot form coacervates at the experimental conditions.

**Figure 4:**
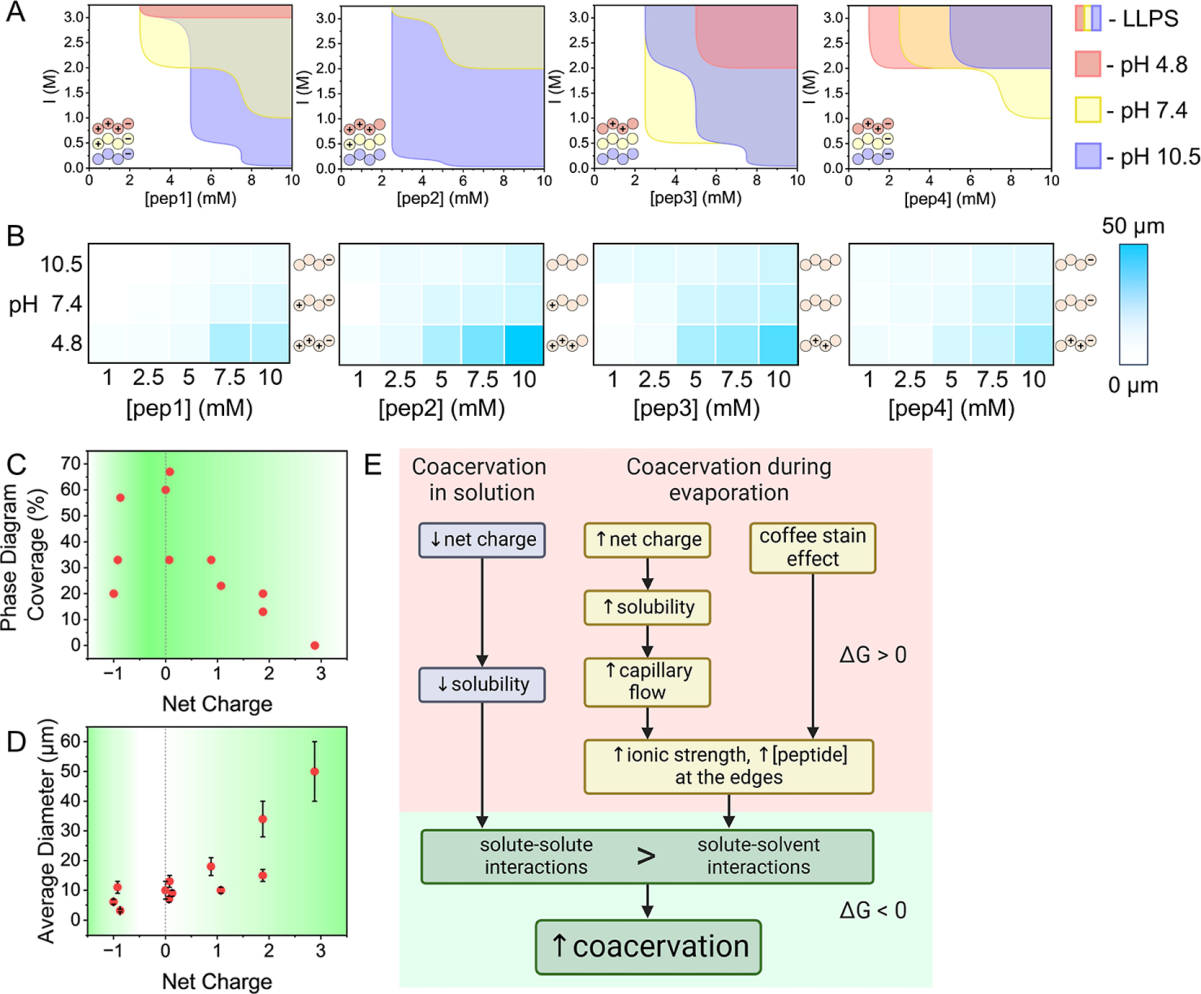
Phase diagrams of the four peptides in the two coacervation processes. (A) Phase diagrams for the four peptides in solution, describing the peptide concentrations and ionic strength values at which coacervates are formed. Insets: the charged groups of each peptide at the corresponding pH value. (B) Phase diagrams for the four peptides in the evaporation process, based on a heat map of the average diameter of the formed coacervates at the center of the deposited droplet. (C) Coverage percentage of the phase diagrams in solution against the net charge values of the corresponding peptides. (D) Average diameter of the coacervates against their corresponding net charge values. The data points and error bars represent the arithmetic mean and standard deviation values of 30 different coacervates for each point. (E) Scheme depicting the thermodynamic considerations that result in coacervation during both processes.

At pH 4.8, out of the four peptides, pep4 can form coacervates at the lowest ionic strength and peptide concentration, while pep2 does not form coacervates at all. This is in accordance with the peptides’ net charge, which is lowest for pep4 (+0.88), intermediate for pep1 and pep3 (+1.88), and highest for pep2 (+2.88). At pH 7.4, pep3 is most capable of forming coacervates due to its low net charge of +0.08, while pep2 is the least capable, with a high net charge of +1.07. On the other hand, pep1, which has a net charge of +0.07, is not as capable as pep3, possibly due to its zwitterionic nature (its absolute net charge is 2.07 compared to 0.08 for pep3) (Figure S7). Comparing pep4 and pep2 at pH 7.4, which have similar absolute net charges (1.08 and 1.07, respectively) but opposite net charges (−0.92 and +1.07, respectively), shows that pep4 can form coacervates at lower ionic strength values. This suggests that there is some preference for coacervation of negatively charged peptides over positively charged peptides. A possible explanation for this could be interactions formed between the larger phosphate ions from the buffer and pep2, which inhibit its condensation into coacervates. At pH 10.5, pep4, which has the most negative charge of the four peptides (−1.00), is the least capable of forming coacervates, as expected. The results of the phase diagrams at the three pH values indicate that the simple coacervation depends on the ionic nature of the peptides, nonionic peptides (pep2, pH 10.5 and pep3, pH 7.4 and 10.5) having the strongest inclination to form coacervates, followed by negatively charged (pep1, pH 10.5 and pep4, pH 7.4 and 10.5), zwitterionic (pep1, pH 4.8 and 7.4 and pep4, pH 4.8), and finally positively charged peptides (pep2, pH 4.8 and 7.4 and pep3, pH 4.8). The difference between the coacervation capabilities of the anionic and cationic peptides could possibly be explained by the spread of charges on the peptides. The negative charge of the anionic peptides is localized to the C-terminus while the positive charges of the cationic peptides can be located at the N-terminus and at both imidazole groups, and as such, they are more dispersed on the peptide.

Figure 4B depicts the sizes of the coacervates obtained with the four peptides at different concentrations and pH values in the process of coacervation through evaporation (Figure S8). The basic assumption in these diagrams is that the peptide’s tendency to form coacervates directly correlates with the extent of coalescence, which results in larger coacervates. In this process, we can observe the opposite phenomenon, as lower pH seems to promote coacervation and results in larger particles. At lower pH values, the peptides obtain higher net charges, increasing their solubility. This could expedite the transport of the free peptides and the smaller coacervates to the droplet’s perimeter due to the outward capillary flow.^17,34^ For example, the peptide with the highest net charge is pep2 at pH 4.8, and it also produced the largest coacervates (50 ± 10 μm). Peptides with a lower solubility due to their lower net charges could precipitate faster and would not take part in the coacervation. The connection between the peptides’ net charge and their ability to coacervate is clear from Figure 4D, which shows that the coacervation capabilities are stronger when the peptides have higher net charges. Additionally, both control peptides AHHA and WAAW do not form coacervates, while the peptides NH_2_-Phe-(His)_2_-Phe-COOH (FHHF) and NH_2_-Tyr-(His)_2_-Tyr-COOH (YHHY) do (Figure S9). This can be explained by the need of a sticker-and-spacer motif that is found only in FHHF and YHHY, while AHHA lacks the hydrophobic stickers and WAAW lacks the hydrophilic spacer.

Each process appears to reach favorable conditions for coacervation by a different route. In solution, a lower net charge decreases the peptides’ solubility, favoring peptide–peptide interactions (Figure 4E). In coacervation during evaporation, peptide molecules can be transported to the edges via the coffee stain effect. Additionally, a higher net charge increases the peptides’ solubility and enhances their transport to the edges with the capillary flow. The high peptide concentration at the edges of the droplet, combined with the high ionic strength there due to evaporation, similarly favors the peptide–peptide interactions that result in coacervation. In both processes, the coacervation may be the result of a negative enthalpy change due to the peptide–peptide interactions, a positive change in entropy due to the exclusion of water molecules from the coacervates, or both.^35^

### Driving Forces for the Coacervation

The coacervates were characterized by using Fourier transform infrared (FT-IR) spectroscopy in solution. The spectrum of free pep1 molecules at pH 10.5 showed several peaks attributed to the carboxylic group stretching vibration at 1407, 1593, 1642, and 1673 cm^–1^ (Figure 5A).^36–40^ When the ionic strength of the solution was increased from 0 to 3 M and the coacervates were formed, the intensity of these peaks was decreased significantly, probably due to the interaction of the carboxylic group with the sodium counterions of the salt. The peaks at 1439, 1447, and 1457 cm^–1^ in the spectrum of the free peptide can be attributed to CH_2_ bending.^41,42^ The peaks at 1439 and 1457 cm^–1^ can be attributed to C–N stretching of the histidine and tryptophan groups, respectively.^42^ Finally, the peak at 3437 cm^–1^ can be attributed to NH stretching of the amine, imidazole, and indole groups.^39,40,43,44^ The intensities of all of these peaks increase when coacervates are formed. This increase can result from the organization of the peptide molecules inside the coacervates that makes the groups associated with these peaks more responsive to infrared radiation. Such a change could occur from the appearance of hydrogen bonds to these NH and C–N groups of the amine, histidine, and tryptophan in the peptide. The intensity increase can also be caused by the coacervation process itself, which condenses a higher concentration of the peptide molecules, increasing their signal.

**Figure 5:**
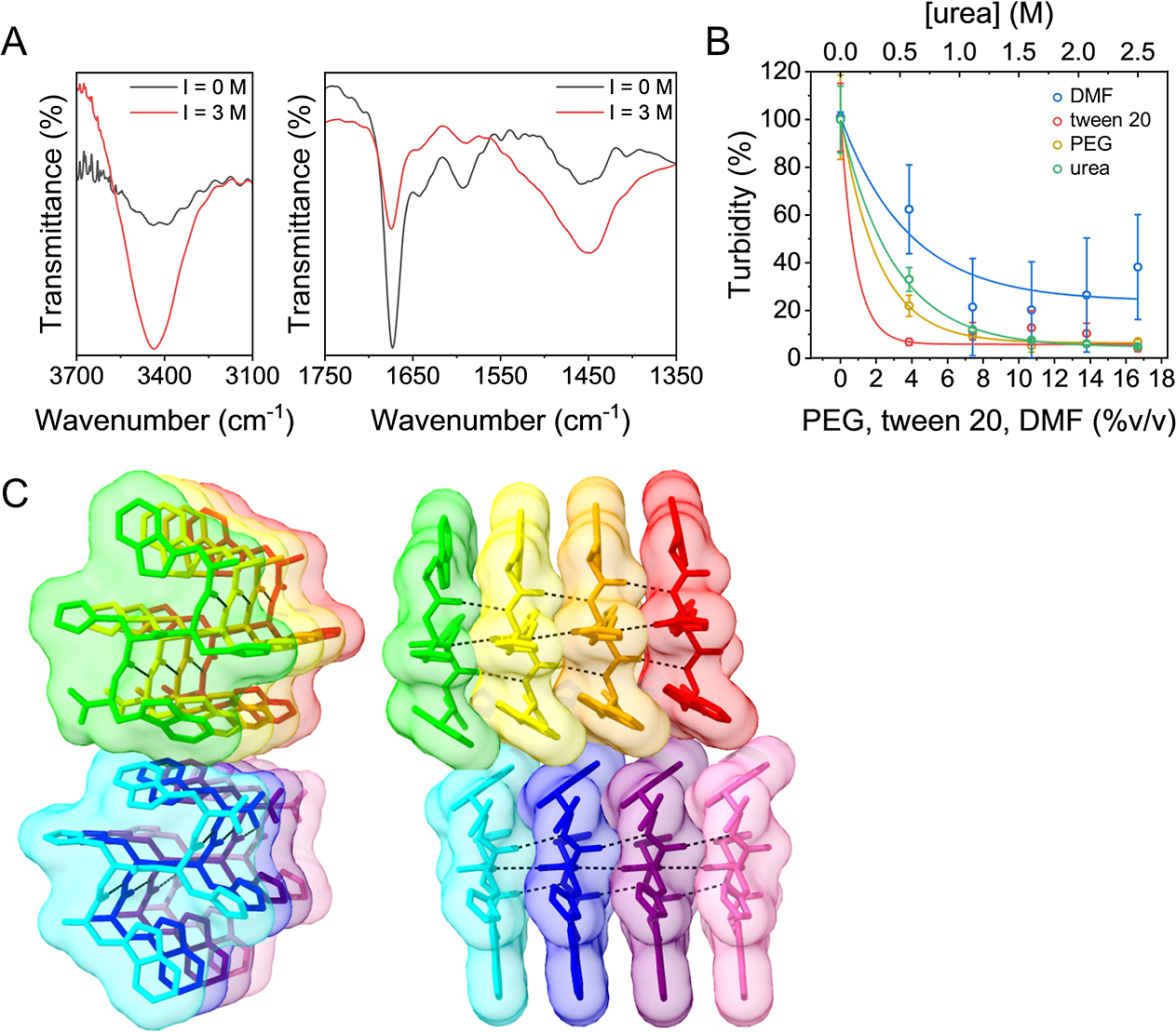
Driving forces of the coacervation. (A) FT-IR spectra of pep1 in a solution with low (black) and high (red) ionic strengths. (B) Turbidity of a solution of pep4 coacervates during titration with DMF, tween 20, PEG, and urea, fitted to an exponential decay equation. The data points and error bars represent the arithmetic mean and standard deviation values of triplicate measurements. (C) Structures of eight pep1 molecules predicted using AlphaFold viewed in two orientations. Hydrogen bonds are displayed as dashed black lines.

To gain insight into the driving forces of coacervation, the coacervates were titrated with different molecules that reveal the important types of interactions for this system. By forming hydrogen bonds with the polar groups of the peptide and dispersion interactions with its apolar and hydrophobic groups, urea and DMF compete with the intra- and intermolecular interactions that the peptide forms with itself, with other peptides, and with the water molecules.^19,45–47^ These titrants can also interfere with π–π interactions.^19,47^ A sharp decrease in the turbidity caused by the coacervates was observed, dropping below 50% at only ∼0.3 M urea and ∼4% (v/v) DMF (Figures 5B and S10). This indicates that hydrogen bonds, π–π stacking, and hydrophobic interactions are crucial for stabilizing the coacervates formed by this peptide. The peptides’ weaker inclination to form coacervates at lower pH values in solution may also hint at the significance of the hydrogen bonds, as the protonation of the N-terminal amine or imidazole groups prevents them from acting as hydrogen bond acceptors, which may decrease the peptides’ coacervation capabilities. Titrating with tween 20, a nonionic surfactant that can interfere with hydrophobic interactions, also revealed the importance of these interactions to the system.^47^ Additionally, a titration with polyethylene glycol (PEG 400) similarly decreased the turbidity, indicating that depletion attraction is not crucial for this system.^48^ The disassembly of the coacervates upon addition of PEG may be the result of them competing for hydrogen bonds, similar to the results with DMF and urea.

Electrostatic interactions do not appear to play a significant role in the coacervation processes. The zwitterionic peptides (pep1 at pH 4.8 or 7.4 and pep4 at pH 4.8), which could benefit from intermolecular electrostatic interactions, do not exhibit better coacervation capabilities than the anionic or nonionic peptides in solution or than the cationic peptides in coacervation during evaporation. Additionally, coacervation could occur at lower concentrations of all four peptides by increasing the ionic strength. This indicates that the screening of the charges of the peptides by the salt promotes coacervation, further implying that electrostatic interactions between the peptide molecules do not act as the driving force behind this process. Similarly, cation–π interactions do not appear to contribute to the coacervation in solution, as the cationic peptides are the least prone to coacervate.

Structures of eight pep1 molecules predicted by AlphaFold^49^ show intermolecular hydrogen bonds that may stabilize the coacervates in accordance with the results from the urea titration (Figure 5C). Additionally, the structures show π–π stacking interactions between the indole groups of the tryptophan. The predicted structures show a resemblance to intermolecular β-sheet structures. This existence of such an organization of the peptide molecules in the coacervates could be supported by the substantial increase in the fluorescence of thioflavin T (ThT) inside the coacervates, which occurs when ThT is in the presence of β-sheets (Figure 6E,F).^50^

**Figure 6:**
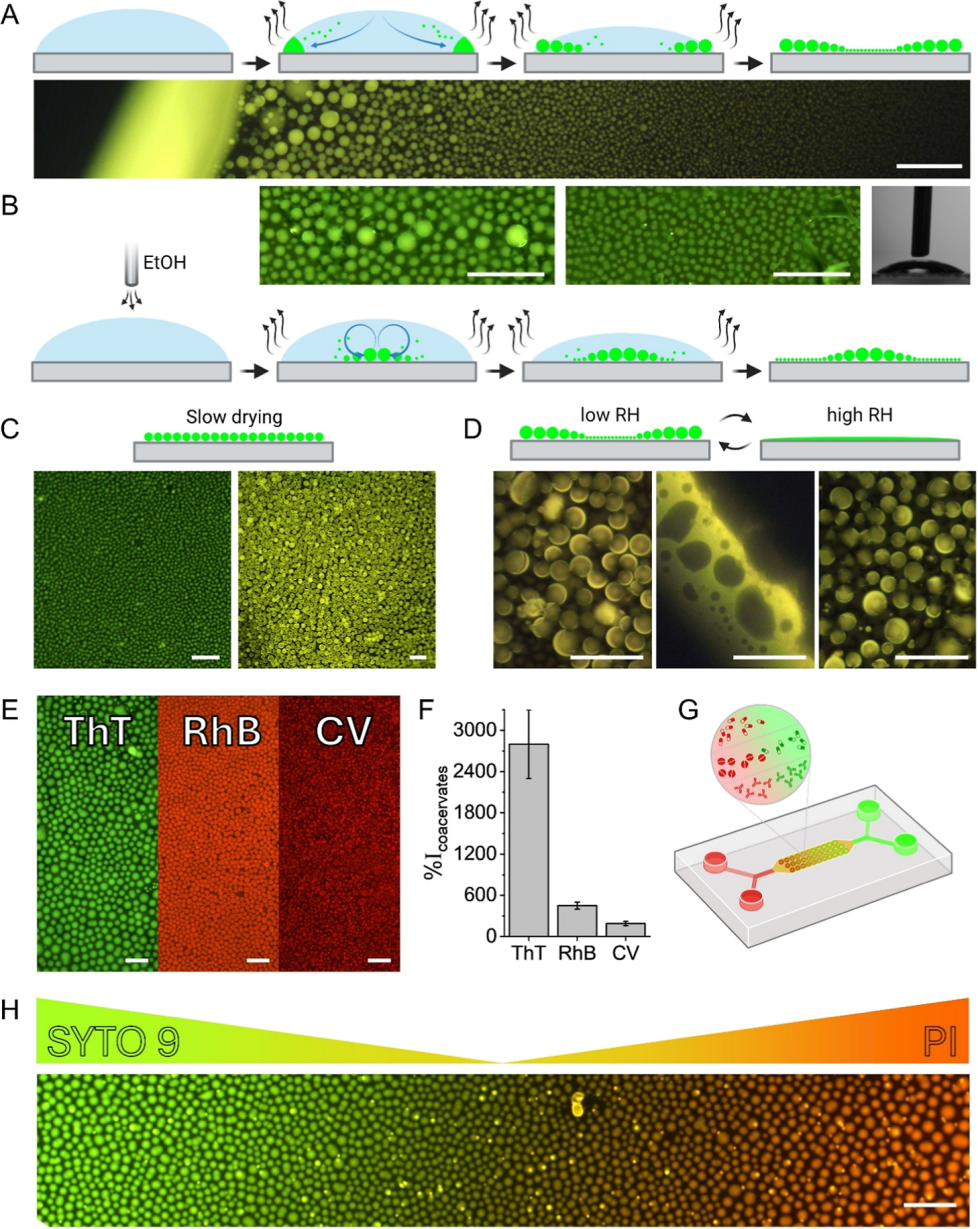
Controlled deposition and encapsulation. (A) Scheme and a fluorescence microscopy image of the size gradient formed by pep1 coacervates during evaporation (scale bar: 50 μm). (B) Scheme and fluorescence microscopy images of the deposition of coacervates of pep1 under an external source of ethanol vapor. The images represent the coacervates congregating under the vapor source (left), away from the source (middle) (scale bars: 50 μm), and an image of the droplet under the vapor source (right). (C) Scheme and fluorescence microscopy images of pep1 coacervates deposited at 4 °C (left) and on a parafilm substrate (right) (scale bars: 50 μm). (D) Scheme and fluorescence microscopy images of pep1 coacervates before (left), during (middle), and after (right) increasing the RH (scale bars: 50 μm). (E) Fluorescence microscopy images of pep2 coacervates formed during evaporation, encapsulating ThT, RhB, and CV (5 mg/L) (scale bars: 50 μm). (F) Fluorescence of ThT, RhB, and CV increases inside the pep2 coacervates. The data points and error bars represent the arithmetic mean and standard deviation values of triplicate measurements. (G) Scheme representing possible uses of a device equipped with the concentration gradients that form inside the coacervates. (H) Fluorescence microscopy images of concentration gradients of the fluorescent dyes SYTO 9 (0.75 μM) and PI (3.75 μM) inside the coacervates of pep1 formed during evaporation (scale bars: 50 μm).

### Controlled Deposition and Encapsulation

The ability to control the deposition of the coacervates formed during evaporation on the substrate was investigated. As previously explained, when allowed to evaporate at room temperature, the capillary forces distribute the coacervates to the perimeter of the droplet, where they coalesce, forming larger and larger particles the closer they get to the edge (Figure 6A and Video S3). By placing the evaporating droplet underneath an external source of ethanol vapors, the Marangoni effect could be enhanced, opposing the capillary flow and flipping the size gradient (Figure 6B). The larger coacervates that appeared underneath the vapor source indicate that the Marangoni flow transported the coacervates to this area, where they coalesced, forming larger coacervates inside the droplet. When deposited on a hydrophobic substrate, the Marangoni flow transported the coacervates to the center of the droplet in the same manner (Figure S11). The coalescence that results in larger coacervates is not distinct on the hydrophobic substrate, probably due to the longer duration of evaporation that allowed the coacervates to solidify before congregating.

The size that the coacervates can reach appeared to be directly affected by the volume of the deposited solution. A droplet of 0.2 μL gave rise to coacervates smaller than 500 nm, while 5 μL provided large coacervates with diameters of several micrometers (Figure S12). Larger deposited volumes also resulted in more hemisphere structures. This size gradient could be eliminated when the droplets are given time to reach equilibrium before complete evaporation of the solution. This was demonstrated when the evaporating droplet was placed at 4 °C or on a hydrophobic substrate (Figure 6C). In these cases, no size gradient was formed, and the size distribution was more homogeneous.

The formed layer of coacervates could be disassembled by increasing the relative humidity (RH) of the substrate’s environment (Figure 6D). The high RH dissolves the coacervates into a thin layer of concentrated peptide solution. When the original RH value is restored, the coacervates reform spontaneously. This behavior remained for five cycles of changing the RH (Figure S13A). This could be advantageous for applications that require the release of encapsulated material under high humidity conditions such as the release of pesticides in agricultural applications. In earlier research, we demonstrated how these peptides can bind certain metal ions.^51^ Mixing the desired metal ions with the peptide coacervates, followed by reducing the metal ions, may increase the stability of the coacervates in solution and prevent their disassembly on the surface at high humidity conditions. Specifically, pep2 and pep3 were shown to bind Fe^2+^ ions that may be suitable for biomedical applications. Encapsulation of certain compounds inside the coacervates can also prevent the disassembly. This was observed for coacervates containing rhodamine B (RhB), where not all coacervate particles disassembled upon increasing the RH (Figure S13B). Alternatively, coating the coacervates with a protective layer, e.g., chitosan, polydopamine, etc., could possibly prevent this disassembly.

Many of the most widely used pesticides are aromatic, such as dichlorodiphenyltrichloroethane (DDT), atrazine, 2,4-dichlorophenoxyacetic acid, chlorothalonil, and azoxystrobin.^52^ Aromatic compounds are also commonly used as various drugs and dietary supplements, for example, 99% out of a data set of 3566 medicinal compounds were found to contain aromatic rings.^53,54^ The ability of the coacervates to encapsulate various aromatic compounds was investigated. The coacervates were shown to successfully encapsulate ThT, RhB, and crystal violet (CV) (Figure 6E). These dyes were chosen specifically for their resemblance to compounds that can be encapsulated inside the coacervates for applications such as drug delivery and biosensing. ThT is a benzothiazole salt. Benzothiazole is used on its own as a food additive, and its derivatives may be found in various drugs such as riluzole, lubeluzole, and ethoxazolamide.^55,56^ On the other hand, RhB and CV are dyes commonly used in the textile industry and as indicators.^57^ Thus, they are relevant for manufacturing sensors. The fluorescence intensity originating from these dyes inside the coacervates was significantly higher than in solutions without the peptide, indicating efficient partitioning (Figure 6F). Both ThT and CV are cationic dyes, while RhB is zwitterionic. Nevertheless, the positive charges of the dyes did not appear to adversely affect their partitioning in the cationic peptide coacervates. The ability of the four peptides to encapsulate RhB was compared at pH 7.4 (Figure S14A). At this pH, pep1 is zwitterionic, pep2 is cationic, pep3 is nonionic, and pep4 is anionic. The nonionic pep3 showed better partitioning capabilities of RhB than the cationic pep2, which is expected due to the repelling cationic charge of RhB. Other than that, the peptides behaved similarly, indicating that the charge screening from the salt ions negates the effects of the charge of the peptides on their ability to encapsulate. This behavior can also be observed when comparing the RhB partitioning ability of pep1 at different pH values. In this case, even though the net charge of the peptide increases when the pH decreases, the partitioning efficiency is increased (Figure S14B). Figure 4B shows that the coacervation capabilities of pep1 during evaporation are greater at lower pH values. These results indicate that the determining factor for the dye partitioning is not the charge of the peptide but its ability to form coacervates, as demonstrated in the phase diagrams.

The stability of the coacervates under various conditions was assessed to confirm their feasibility for drug delivery. Due to its ability to form coacervates at the three pH values tested and at relatively lower ionic strength values (Figure 4A), pep3 was chosen for these measurements. At pH 10.5, the coacervates appear to flocculate after 1.6 h, possibly due to the nonionic nature of the peptide at this pH (Figure S15A). The flocculation indicates that the coacervates solidify. Nonetheless, the particles remain in the solution for >68.4 h. At pH 7.4, the coacervates remained stable for up to 21.9 h, while at 4.8, they remained up to 2.5 h. The correlation between the stability and the pH may be explained by the higher solubility of the peptide at acidic pH values due to its higher net charge. In fetal bovine serum (FBS), no coacervates were visible after 21.9 h, similar to the results in the phosphate buffer. This pH-dependent behavior may be suitable for injection of drugs for circulation in the serum, or for a targeted release near tumors due to their lower extracellular pH.^58^ The encapsulation of RhB inside the coacervates at pH 7.4 appeared to stabilize them, and they remained in the solution even after 68.4 h. This is similar to the disassembly of RhB-containing coacervates at high RH (Figure S13B). However, at pH 4.8, the coacervates disassembled and released encapsulated RhB after 3.7 h. An increase in the fluorescence intensity from the RhB for the coacervates at pH 7.4 can be observed. This points to the solidification of the coacervates that increases the fluorescence due to restricted molecular motion, similar to what may occur for ThT.^15^ On the other hand, at pH 4.8, there appears to be no fluorescence increase, indicating that the coacervates remain more liquid-like and can disassemble more readily. The deposited coacervates formed during evaporation remained stable on the surface for at least 1 week (Figure S15B).

A unique deposition that can be achieved in coacervation during evaporation is the arrangement of gradients of dyes in the coacervates (Figure 6G). By adding single or multiple dyes on top of the evaporating droplet with the peptide, the coacervates that form can encapsulate the dyes at differing concentrations. This can be used to form surface gradients of the single dye or gradients of combinations of dyes in ways that were previously impossible (Figures 6H and S16). This special deposition method may allow to fabricate gradients of indicators that can be used as sensors with higher sensitivity. Forming gradients of drug molecules may aid in research focused on finding the optimal drug dosage for specific cells or optimizing synergistic or antagonistic drug combinations. The gradients could also be used to study antibiotic resistance by probing the response of different bacteria to various concentrations of antibiotics.^59^ Additionally, it may be possible to utilize these gradients in dye-sensitized solar cells, photonic devices, and metamaterials.

### Antioxidant Activity

The ability of the coacervates to encapsulate curcumin was assessed. Curcumin is a compound that is commonly taken as a dietary supplement due to its health benefits and low bioavailability from food sources.^60^ To increase its bioavailability, curcumin can be administered in emulsions or along with piperine, a compound that blocks its metabolism in the body. We investigated the ability of the coacervates to encapsulate curcumin, as well as piperine. Curcumin was shown to be effectively encapsulated in the coacervates with a high encapsulation efficiency percentage (% EE) of 96 ± 3% due to its low water solubility (Figure S17). This efficiency is in the range of other encapsulation systems for curcumin, such as particles, liposomes, and other coacervates (Table S1). Piperine, which has one less aromatic ring and is ∼26 times more water-soluble than curcumin according to the ALOGPS software,^61^ was encapsulated less effectively with an % EE value of 41 ± 6%. While this value is lower than other encapsulation systems for piperine (Table S1), it can still increase the bioavailability of curcumin. Overall, these values indicate that the peptide coacervates could serve as an effective vehicle for curcumin as a dietary supplement.

The effect of the encapsulation of curcumin on its stability was investigated through its absorbance. Alkaline pH causes rapid degradation of curcumin, initially forming an enol form that later degrades to compounds such as vanillin and ferulic acid.^62^ UV irradiation can cause curcumin degradation regardless of the solution’s pH. These processes can be observed in Figure 7A. When curcumin was encapsulated inside the coacervates, alkaline pH as well as exposure to direct UV light for 3 h did not cause significant degradation. This demonstrates the advantage of using the coacervates as delivery vehicles for dietary supplements as they can protect the encapsulated compounds.

**Figure 7:**
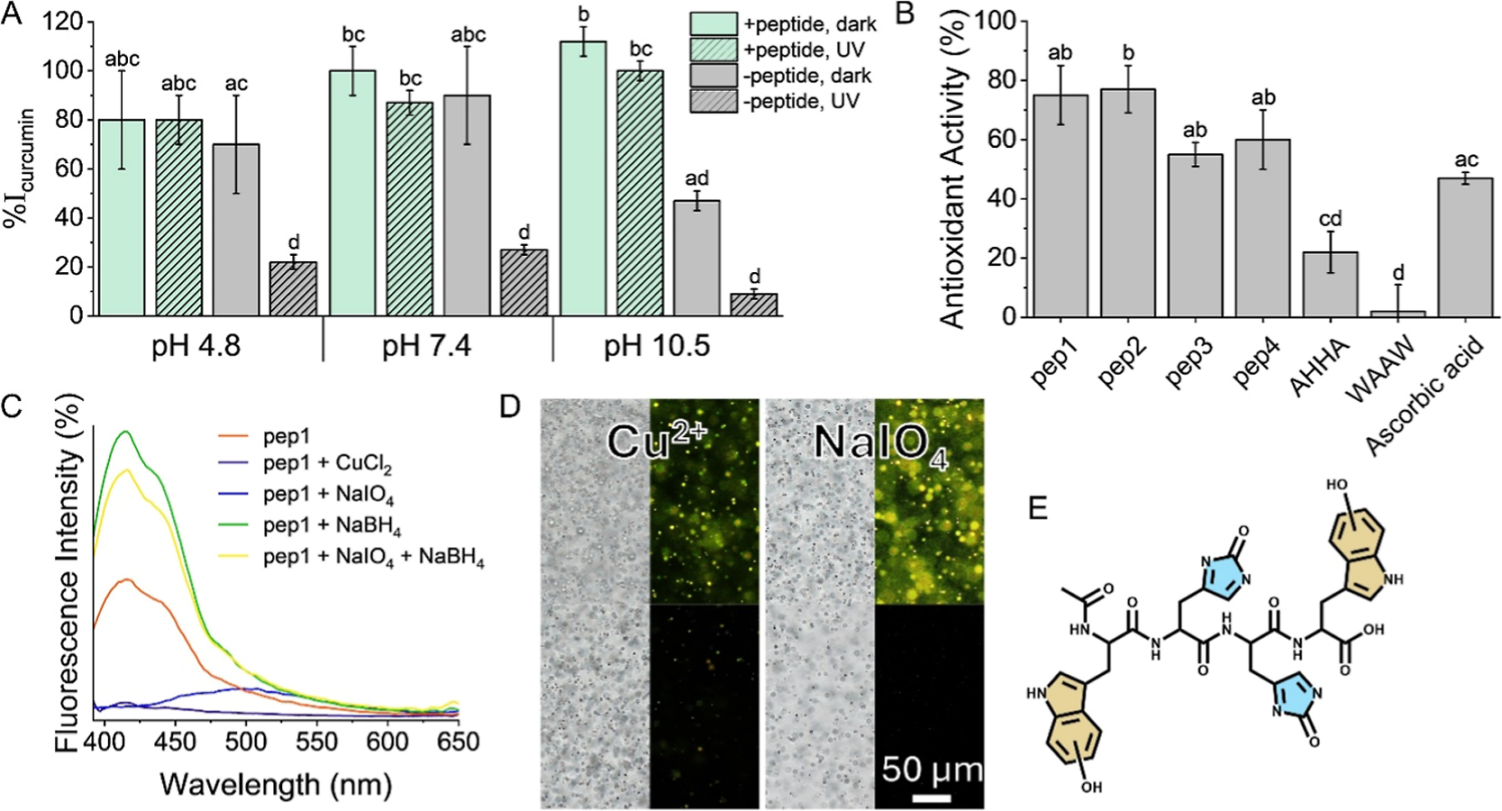
Antioxidant properties of the peptides. (A) Absorbance intensity of curcumin (50 mg/L) encapsulated inside pep4 coacervates formed in solution (green) or free curcumin in solution (gray) at different pH values with (slanted lines pattern) or without (no pattern) exposure to UV irradiation, relative to the intensity of fresh curcumin at the corresponding pH. (B) DPPH assay results for different peptides and ascorbic acid (8 mM). The data points and error bars represent the arithmetic mean and standard deviation values of triplicate measurements. The results were compared using a one-way ANOVA test with Tukey–Kramer post hoc analysis. Statistically significant results were determined at *p* < 0.05 and grouped with letters. (C) Fluorescence intensity percentage of pep1 in oxidized or reduced states. (D) Transmitted light and fluorescence microscopy images of pep4 coacervates before (above) and after (below) mixing with CuCl_2_ (left) or NaIO_4_ (right). (E) Chemical structure of an oxidized form of pep4.

To derive the origin for the peptides’ ability to protect curcumin from degradation, we performed a 2,2-diphenyl-1-picrylhydrazyl (DPPH) assay that determines the antioxidant activity of a compound. The assay revealed that all four peptides have strong antioxidant capabilities (Figure 7B). This antioxidant activity was also observed when the four peptides were mixed with divalent cupric ions for 30 min. The peptides reduced the copper ions to obtain monovalent cuprous ions (Figure S18). This conversion appeared to be more effective when pep3 and pep4 were used, possibly due to the lack of an amine group that could interact with the copper ions, protecting them from the reduction. The same reduction capabilities were observed when the peptide reduced Au^3+^ and Ag^+^ ions (Figure S19).

The reduction of the metal ions and the corresponding oxidation of the peptides diminish their fluorescence (Figure 7C). The same occurs when the peptides were mixed with NaIO_4_, which is a strong oxidizing agent. The fluorescence was retrieved by reducing the peptides with NaBH_4_. On the other hand, the oxidation of the peptides did not seem to significantly harm their coacervation (Figure 7D). Mass spectrometry was used to determine the oxidized form of the peptide, and a peak that corresponds to a peptide oxidized at both its indole groups and both imidazole groups was obtained (Figures 7E and S20). Overall, these results indicate that the origin of the antioxidant capabilities of the peptides lies in their ability to oxidize their four functional groups. Such a property could be greatly beneficial as a delivery vehicle for dietary supplements as well as certain drugs that can be easily oxidized. Along with their UV-protective capabilities, encapsulation capabilities, intrinsic fluorescence, and the ability to control their deposition, this coacervation system could provide valuable means for delivering various compounds. A summary of the various data that was acquired for the four peptides is provided in Table 1, intended to simplify the use of these peptides for the desired application.

**Table 1:** Table Comparing the Various Results for Each Peptide Obtained in This Research

	pH	net charge	|net charge|	antioxidant activity (%)	cell viability (%)^a^
pep1	10.5	–0.87	1.13		
	7.4	+0.07	2.07	75 ± 10	104 ± 7
	4.8	+1.88	3.88		
pep2	10.5	+0.13	0.13		
	7.4	+1.07	1.07	77 ± 8	99 ± 6
	4.8	+2.88	2.88		
pep3	10.5	0.00	0.00		
	7.4	+0.08	0.08	55 ± 4	95 ± 5
	4.8	+1.88	1.88		
pep4	10.5	–1.00	1.00		
	7.4	–0.92	1.08	60 ± 10	108 ± 6
	4.8	+0.88	2.88		

	solution				evaporation	
	pH	phase diagram coverage (%)	*I*_min_ (M)	stability (h)	average diameter (μm)	stability (h)
pep1	10.5	57	0.05		3.2 ± 0.4	
	7.4	33	1		7 ± 1	
	4.8	13	3		15 ± 2	
pep2	10.5	77	0.05		9 ± 1	
	7.4	23	2		10 ± 1	
	4.8	0			50 ± 10	
pep3	10.5	60	0.05	>68.4^b^	10 ± 3	>167.4
	7.4	67	0.05	21.9	13 ± 2	>167.4
	4.8	20	2	2.5	34 ± 6	>167.4
pep4	10.5	20	2		6.1 ± 0.8	
	7.4	33	1		11 ± 2	
	4.8	33	2		18 ± 3	

a The cell viability measurements were performed in a medium containing 88% RPMI-1640 (pH 7.0–7.4). The displayed results are for the 5 mM concentration of the peptides.

b The coacervates flocculate after 1.6 h.

## Conclusions

In this work, we describe the coacervation capabilities of four intrinsically fluorescent peptide derivatives in two processes: in solution and during evaporation. The effects of the peptides’ net charges at different pH values on their ability to coacervate were investigated. Unique hemispherical structures were obtained, and the process of their formation was analyzed via cryo-SEM. The resulting coacervates displayed efficient encapsulation capabilities of various aromatic compounds. The deposition of the coacervates could be controlled to display either a uniform size of the coacervates, a size gradient, or a concentration gradient of the encapsulated material. Lastly, the peptides and resulting coacervates exhibit strong antioxidant capabilities that can protect the encapsulated material.

Since the peptides are short and made from natural amino acids, they can be relatively cheap to synthesize. Additionally, the preparation of the coacervates in both processes is simple and requires no instrumentation or additional compounds other than the peptide and the salt. By introducing the peptides into the appropriate environment as described in this research, the coacervates form spontaneously. If the targeted outcome is deposition on a hydrophilic surface, this preparation is especially feasible, as a relatively small volume can be sufficient to cover the surface.

Overall, the coacervates investigated in this work show significant advantages for delivering and protecting various compounds, such as dietary supplements. The ability to specifically design the required coacervate particles through altering different parameters such as the peptide’s end groups, coacervation process, pH, ionic strength, deposition process, and encapsulated material concentration contributes to the potential of this particle-forming system. The knowledge of how these parameters affect the resulting coacervates can help with matching the coacervates to the conditions of the desired application. For example, if there is a need to form stable coacervates under acidic conditions, pep4 might be the best option; under alkaline conditions, pep2, etc.

The ability of these peptides to bind metal ions may also be used in the future to provide coacervates with unique properties from the metal. Additionally, it is intriguing to study whether the fluorescence of these peptides may allow them to be used in some form as fluorescent tags for proteins, which may also increase their ability to coacervate.

## Experimental Section

### Materials

2-Chlorotrityl chloride resin (1.0–1.6 mmol/g, 100–200 mesh) was purchased from Chem-Impex International (Wood Dale, IL, United States). 1-[Bis­(dimethylamino)­methylene]-1*H*-1,2,3-triazolo­[4,5-*b*] pyridinium 3-oxide hexafluorophosphate (HATU) and Rink amide resin (0.48 mmol/g) were purchased from Matrix Innovation (Saint-Hubert, QC, Canada). Triisopropylsilane (TIPS) and Nile red were purchased from the Tokyo Chemical Industry (Tokyo, Japan). Dimethylformamide (DMF), dichloromethane (DCM), piperidine, methanol, diethyl ether, *N*,*N*-diisopropylethylamine (DIEA), acetonitrile (ACN), chloroform, acetic acid, sodium bicarbonate, and trifluoroacetic acid (TFA) were purchased from Bio-Lab (Jerusalem, Israel). Fmoc-Trp­(Boc)-OH and peptides FHHF, YHHY, AHHA, and WAAW were purchased from GL Biochem (Shanghai, China). Hydrogen tetrachloroaurate­(III) hydrate, sodium borohydride, and copper­(II) chloride were purchased from Acros Organics (Fair Lawn, NJ, United States). Silver nitrate 99+%, crystal violet, sodium ascorbate, and Fmoc-His­(Trt)-OH were purchased from Alfa Aesar (Lancashire, United Kingdom). NaCl was purchased from Fisher Scientific (Hampton, NH, United States). Rhodamine B, SYTO 9, propidium iodide, curcumin, tween 20, and piperine were purchased from Thermo Fisher Scientific (Waltham, MA, United States). Sodium dodecyl sulfate (SDS) was purchased from J.T. Baker Inc. (Phillipsburg, NJ, United States). Rhodamine 110 chloride, urea, thioflavin T, PEG 400, sodium acetate, sodium periodate, and sodium phosphate dibasic heptahydrate were purchased from Merck (Darmstadt, Germany). Acetic anhydride and sodium carbonate were purchased from Daejung (Siheung-si, South Korea). Sodium phosphate monobasic monohydrate was purchased from Mallinckrodt Pharmaceuticals (Dublin, Ireland). A pierce bicinchoninic acid (BCA) protein assay kit was purchased from Thermo Fisher Scientific (Waltham, MA, United States). Fetal bovine serum was purchased from Biowest (Nuaillé, France).

### Peptide Synthesis

The four investigated peptides were manually synthesized by the Fmoc solid phase peptide synthesis. Pep1 and pep4 were synthesized on a 2-chlorotrityl chloride resin, while pep2 and pep3 were synthesized on rink amide resin (0.25 mmol each). The amino acids (5 equiv) were activated by mixing with a DIEA/HATU mixture (4 and 3.9 equiv, respectively) for 4 min. The coupling of the amino acids was carried out for 1 h and confirmed by using a Kaiser test. The Fmoc protecting groups were removed by using 20% piperidine in DMF for 20 min. The resin was washed between steps twice with DMF, methanol, and DCM, and again with DMF. For pep2 and pep3, acetylation was performed by mixing the resin in a DMF solution with acetic anhydride and DIEA (50 equiv each) for 30 min. The capping was also confirmed by using a Kaiser test. The cleavage reaction was performed by mixing it with a TFA/TIPS/water mixture (38:1:1) for 3 h. The cleaved solution was evaporated, precipitated with diethyl ether, and centrifuged. The peptide product was dissolved in water and lyophilized.

The purity of the peptides was assessed using analytical reverse-phase high-performance liquid chromatography (HPLC) analysis (Waters Alliance) with UV detection (220 and 280 nm) and an XSelect C18 column (3.5 mm, 130 A°, 4.6 mm 3150 mm). The peptides were eluted using a linear gradient of ACN in triply distilled water (TDW) (0.1% TFA, 1 mL/min, 30 °C). The mass of pep1 was determined using liquid chromatography mass spectrometry (LC/MS) with an Agilent 6520 Q-TOF analyzer (Agilent Technologies, Santa Clara, CA, United States). The masses of pep2, pep3, and pep4 were determined by using MALDI TOF/TOF AutoFlex Speed (Bruker Daltonics, Bremen, Germany).

### Net Charge Calculations

The net charge values of the peptides at pH 4.8, 7.4, and 10.5 was determined using the equation: $Z=\sum_{i}N_{i}\frac{10^{pK_{a,i}}}{10^{pH}+10^{pK_{a,i}}}-\sum_{j}N_{j}\frac{10^{pH}}{10^{pH}+10^{pK_{a,j}}}$ , where *Z* is the net charge, *N*_*i*_ is the number of positively charged groups in the peptide, i.e., the histidines and the N-terminus, depending on the peptide, *N*_*j*_ is the number of negatively charged groups, i.e., the C-terminus, and p*K*_a,*i*_ or p*K*_a,*j*_ are their corresponding p*K*_a_ values.^63^ The absolute net charge was calculated by adding the two components to the equation rather than subtracting them.

### Coacervation Processes and Phase Diagrams

The coacervation was performed in two different processes. In general, when performed in solution, the peptide was added into a buffer solution (25 mM) to achieve the desired concentration with the desired ionic strength using NaCl. The buffers used were acetate buffer (pH 4.8), phosphate (pH 7.4), and carbonate (pH 10.5). For the coacervation during evaporation, the peptide solution was added to the desired buffer solution (with 0.3 M NaCl). Triply distilled water was added when necessary to achieve the same concentrations between samples. Droplets (2 μL) were deposited on clean glass slides (76 mm × 26 mm × 1 mm, Paul Marienfeld GmbH & CO. KG, Lauda-Königshofen, Germany) and viewed using light or fluorescence microscopy (Axio Scope A1 fluorescence microscope, Zeiss, Oberkochen, Germany). For the coacervates prepared in solution, the images were taken immediately after depositing the solution on the slide, to avoid the formation of evaporation-induced coacervates. The glass slides were cleaned before use by treatment with UV/ozone (Jelight, Irvine, California, United States) for 10 min, submerging in SDS (2%, 30 min), and treating with O_2_ plasma (Atto, Diener Electronic, Ebhausen, Germany) for 1 min. All measurements for the phase diagrams were performed in triplicate.

The coverage of the phase diagram was calculated by determining the ratio between the sampled conditions that resulted in LLPS and the overall sampled conditions. The average diameter of the coacervates from the phase diagram of the evaporation process was calculated using the ZEN microscopy software (Zeiss, Oberkochen, Germany) by averaging the diameters of 30 coacervates that represent the most common size in the sample and are not located at the perimeter of the deposited droplet to avoid the influence of the size gradient.

### Scanning Electron Microscopy

High-resolution scanning electron microscopy (SEM) images were obtained using a Magellan 400L (Thermo Fisher Scientific), operating at 2 kV. The sample of the coacervates in solution was prepared with pep4 (5 mM) in acetate buffer (pH 4.8, 25 mM, 3 M NaCl). The samples of the coacervates that form during evaporation were prepared using pep1 (10 mM) in phosphate buffer (pH 7.4, 25 mM, 0.3 M NaCl). The samples were deposited on clean silicon surfaces.

The cryo-SEM images were taken by using Apreo 2 S LoVac (Thermo Fisher Scientific). The cryo-SEM sample of the coacervates formed during evaporation was prepared by depositing a droplet (2 μL) of pep4 (10 mM) in acetate buffer (pH 4.8, 25 mM, and 0.3 M NaCl). The droplet was allowed to evaporate for 9 min before being immersed in liquid nitrogen. The sample was then sublimated (85 K, 25 min) before imaging. The sample of the coacervates in solution was prepared by placing two cylinders with a solution of pep4 (7.5 mM) in phosphate buffer (pH 7.4, 25 mM, and 3 M NaCl). The solution was then submerged in liquid nitrogen, and then the two cylinders were separated, fracturing the surface of the frozen droplet. The sample was sublimated before being imaged (85 K, 10 min).

### Absorbance and Fluorescence Measurements

The fluorescence intensity percentage of the peptides were performed by preparing solutions of the peptides (0.1 mM) in phosphate buffer (pH 7.4, 1 mM). The fluorescence intensity percentage of the oxidized or reduced state of pep1 was determined by preparing solutions of pep1 (0.1 mM) mixed accordingly with CuCl_2_, NaIO_4_, and NaBH_4_ (0.1 mM). The excitation wavelength in all measurements was set to 365 nm. All measurements were performed by using a Biotek Synergy H1 plate reader (Lumitron). The results were compared using a one-way analysis of variance (ANOVA) test with Tukey–Kramer post hoc analysis. Statistically significant results were marked with an asterisk.

### FRAP Measurements

The FRAP measurements were conducted on a Nikon Confocal A1R+. The sample was prepared in acetate buffer (pH 4.8, 25 mM, 3 M NaCl) with pep4 (7.5 mM). The measurement utilized the inherent fluorescence of the peptide. The results are an average of eight different coacervates that were bleached with a 405 nm laser, and their fluorescence recovery was probed.

### Atomic Force Microscopy

The atomic force microscopy (AFM) images were taken using a NanoWizard 3 instrument (JPK, Berlin, Germany) at AC mode. The silicon tip used had a spring constant of 6 N/m (Aspire, Team Nanotec GmbH, Villingen-Schwenningen, Germany). The sample of coacervates formed in solution was prepared with pep4 (10 mM) in an acetate buffer (pH 4.8, 25 mM, 3 M NaCl). For the coacervates formed during evaporation, a sample of pep1 (10 mM) in phosphate buffer (pH 7.4, 25 mM, and 0.3 M NaCl) was prepared. In both cases, the solutions were deposited on glass slides and allowed to evaporate before the imaging.

### FT-IR Spectroscopy

The Fourier-transform infrared (FT-IR) measurements were performed in solution by preparing samples of pep1 (10 mM) in D_2_O. A solution of NaOH dissolved in D_2_O was added to increase the pH to ∼10.5. NaCl (3 M) was added to one of the samples. The solution (20 μL) was placed between two CaF_2_ plates by using a spacer (56 μm). The infrared spectra were recorded (2000 scans, 4 cm^–1^ resolution) on a Nicolet 6700 FT-IR spectrometer with a deuterated triglycine sulfate detector (Thermo Fisher Scientific, Waltham, MA, United States). The absorbance peaks were determined by using the OMNIC analysis software (Nicolet).

### Titration Experiments

The titrations were performed by adding aliquots of urea, DMF, PEG, or tween 20 to coacervates of pep4 (5 mM) in phosphate buffer (pH 7.4, 25 mM, and 3 M NaCl). The turbidity values of the samples were measured at 600 nm. The experiment was conducted in triplicate. The data points were fitted to an exponential decay equation.

### Peptide Structure Prediction

AlphaFold v3.0 was used to predict the potential structure of pep1. The algorithm generated 5 structures, and the most optimal model with a predicted local distance difference test (pLDDT) value of ∼80 was displayed (date of modeling: 07/18/2024). The model was verified using Procheck.^64^ The hydrogen bonding in the structures was displayed using ChimeraX.

### Dye Encapsulation

The values of the fluorescence increase inside the coacervates (% *I*_coacervates_) of ThT, RhB, and CV were measured by preparing coacervates of pep2 (5 mM) in acetate buffer (pH 4.8, 25 mM, 0.3 M NaCl) with the dyes (5 mg/L). The % *I*_coacervates_ values for the four peptides were measured using samples containing RhB (5 mg/L) in phosphate buffer (pH 7.4, 25 mM, and 0.3 M NaCl). The % *I*_coacervates_ values at different pH values were measured using samples containing pep1 (5 mM) and RhB (5 mg/L) in the desired buffer (acetate at pH 4.8, phosphate at pH 7.4, or carbonate at pH 10.5, 25 mM, and 0.3 M NaCl). The % *I*_coacervates_ values were calculated according to the equation: $\%{\;I}_{coacervates}=\frac{I_{dye+peptide}-I_{peptide}}{I_{dye}}\times 100\%$ , where *I*_dye+peptide_ is the maximum fluorescence intensity from the coacervates that contain the dye, *I*_peptide_ and *I*_dye_ are the maximum fluorescence intensity of a peptide or dye solutions at the same concentration. The experiments were performed in triplicate, and the results were compared using a one-way ANOVA test with Tukey–Kramer post hoc analysis.

The samples of the enhanced Marangoni flow were prepared by depositing droplets of pep1 (5 mM) in acetate buffer (pH 4.8, 25 mM, 0.3 M NaCl) on a parafilm substrate or on a glass slide underneath a syringe filled with ethanol and capped with a metal needle (0.7 mm diameter). As the volume decreased, the needle was lowered to keep a relatively constant distance from the solution’s interface.

The concentration gradient of SYTO 9 and PI was obtained by depositing a sample (2 μL) of pep1 (10 mM) in phosphate buffer (pH 7.4, 25 mM, 0.2 M NaCl) on a glass slide, adding SYTO 9 (0.2 μL, 0.75 μM) and PI (0.2 μL, 3.75 μM) on opposite sides of the droplet, and allowing the droplet to evaporate while coacervates form.

### Stability Measurements

The stability of the coacervates prepared in solution was examined using coacervates of pep3 (5 mM) in different buffer solutions (25 mM, 3 M NaCl). The coacervates encapsulating RhB (5 mg/L) were prepared using either phosphate or acetate buffers. For the stability in FBS, coacervates of pep3 were prepared in carbonate buffer (pH 10.5, 25 mM, 3 M NaCl), allowed to solidify for 1 h, and diluted in FBS (×20). The stability of the coacervates prepared during evaporation was examined using coacervates of pep3 (10 mM) in different buffer solutions (25 mM, 0.3 M NaCl), deposited on a glass slide.

To observe the RH effects, coacervates of pep1 (10 mM) in phosphate buffer (pH 7.4, 25 mM, and 0.3 M NaCl) were prepared by evaporation on a glass slide. Coacervates were also prepared with RhB (5 mg/L). To increase the RH, the glass slide was placed on a raised platform inside a Petri dish with a TDW for 15 min.

### Curcumin Encapsulation

The encapsulation efficiency values were calculated by using analytical HPLC measurements. Coacervate solutions of pep1 (5 mM) in carbonate buffer (pH 10.5, 25 mM, and 3 M NaCl) with curcumin and piperine (0.3 mM, dissolved in ethanol) were prepared. The solutions were diluted in water (1.5 mM pep1, 0.09 mM curcumin, and piperine) before the HPLC measurements at 343 nm for piperine and 425 nm for curcumin. The experiments were conducted in triplicate.

The absorbance intensity of free or encapsulated curcumin was measured using a Biotek Synergy H1 plate reader (Lumitron). The samples were prepared with pep4 (10 mM) in a buffer solution (25 mM, 3 M NaCl) and curcumin (50 mg/L). The control samples were prepared in the same buffer solution without the peptide. Part of the samples were exposed to UV irradiation for 3 h, followed by dilution of the samples (4 μL) in ethanol (21 μL) and TDW (5 μL) in order to disassemble the coacervates. The absorbance of all samples was measured at 425 nm for samples prepared in acetate and phosphate buffers or at 440 nm for the samples prepared in carbonate buffer due to the red-shifted absorbance of the enol form of curcumin. The experiments were conducted in triplicate, and the results were compared using the one-way ANOVA test with Tukey–Kramer post hoc analysis.

### Cytotoxicity Measurements

The cytotoxicity was measured on human ovarian A2780 cancer cells (purchased from ECACC Inc.), using the MTT assay.^65^ The cells (0.6 × 10^6^) in medium (containing 88% RPMI-1640, 10% FBS, 1% l-glutamine, and 1% penicillin–streptomycin; all purchased from Sartorius) were seeded in 96-well plates in the medium and allowed to attach for 1 day. The cells were subsequently administered with the peptides tested at 10 different concentrations (0.009–5 mM). After 3 days of incubation at 37 °C in 5% CO_2_ atmosphere, MTT (0.1 mg in 20 μL) was added, and the cells were incubated for an additional 3 h. Thereafter, the MTT solution was removed, and 200 μL of isopropanol was added. The absorbance at 550 nm was measured using a Spark 10 M Multimode Microplate Reader spectrophotometer (Tecan Group Ltd.). Each measurement was repeated 3 × 3 times, on different days. The graph was built by GraphPad Prism 5.04 software.

### Antioxidant Assays

The antioxidant activity of the peptides was assessed using DPPH. Peptide solutions (0.1 or 8 mM) in phosphate buffer (pH 7.4, 25 mM) were mixed with DPPH (160 mg/L). Ascorbic acid was used as a control. The mixtures were left for 30 min in a dark place before the absorbance of the DPPH radical at 517 nm was measured using a Biotek Synergy H1 plate reader (Lumitron). The experiment was conducted in triplicate, and the results were compared using a one-way ANOVA test with Tukey–Kramer post hoc analysis.

The reduction of cupric ions by the peptides was followed by using the Pierce BCA protein assay kit. The peptides (5 mM) in carbonate buffer (pH 10.5, 25 mM) were mixed with CuCl_2_ (5 mM). The kit’s reagents A and B were mixed at a 50:1 ratio, and the peptide samples (4 μL) were mixed with the reagents (32 μL). The solutions were left for 30 min before measuring the absorbance of BCA-Cu^+^ at 562 nm using a Biotek Synergy H1 plate reader (Lumitron).

The transmitted light and fluorescence images depicted in Figure 7D were prepared with pep4 (5 mM) in acetate buffer (pH 4.8, 25 mM, 3 M NaCl) before and after the addition of NaIO_4_ or CuCl_2_ (2 equiv).

The mass of the oxidized form of pep4 was found by mixing the peptide (0.2 mM) with CuCl_2_ (1 mM) in water and using LC/MS with an Agilent 6520 Q-TOF analyzer (Agilent Technologies, Santa Clara, CA, United States).

## Supplementary Material









## Notes

Comparison of encapsulation efficiency values; additional fluorescence intensity measurements; additional SEM and cryo-SEM images; additional transmitted light and fluorescence microscopy images; absolute net charge values of the four peptides; additional fluorescence intensity measurements of RhB in the coacervates; encapsulation efficiency values for curcumin and piperine, SEM and fluorescence microscopy images of curcumin-loaded coacervates; absorbance spectra of the peptides in the presence of CuCl_2_ and bicinchoninic acid; and MS analyses and HPLC chromatograms of the peptides (PDF)
Capillary flow in the coacervation during evaporation (MP4)
Coalescence of the coacervates formed during evaporation (MP4)
Size gradient formed by the coacervates during evaporation (MP4)
