# Supplementary material for: Tailoring Peptide Coacervates for Advanced Biotechnological Applications: Enhancing Control, Encapsulation, and Antioxidant Properties
Source: ACS Appl Mater Interfaces. 2025 Apr 28;17(21):31561–74. doi: 10.1021/acsami.5c02367 (PMC12123622; doi:10.1021/acsami.5c02367)
Supplement: Supplementary file 1 [file am5c02367_si_001.pdf]

Supporting Information

Tailoring Peptide Coacervates for Advanced Biotechnological Applications: Enhancing Control, Encapsulation, and Antioxidant Properties

Daniel Boas[1,2], Mohammad Taha[1], Edit Y. Tshuva[1], Meital Reches[1,2,]*

[1]Institute of Chemistry, The Hebrew University of Jerusalem, Jerusalem, 9190401, Israel

[2]The Center for Nanoscience and Nanotechnology, The Hebrew University of Jerusalem, Jerusalem, 9190401, Israel

* Email: meital.reches@mail.huji.ac.il

Video S1. Capillary flow in the coacervation during evaporation.

Video S2. Coalescence of the coacervates formed during evaporation.

Video S3. Size gradient formed by the coacervates during evaporation.

Table S1. Comparison of the encapsulation efficiency values of various encapsulation systems for curcumin and piperine.

Encapsulation System		%EE	Reference
Curcumin	Peptide coacervates	96	this work
	Zein-gum arabic-tannic acid composite particles	93.32	1
	Casein-pectin nanocomplexes	93	2
	Gum arabic and whey protein nanofibrils coacervates	98.88	3
	CTAB micelle-embedded silica particles	>99	4
	liposomes	98	5
Piperine	Peptide coacervates	41	this work
	Polycaprolactone nanoparticles	84.8	6
	Nanoemulsions	89.32	7
	Nanoparticles	99	8

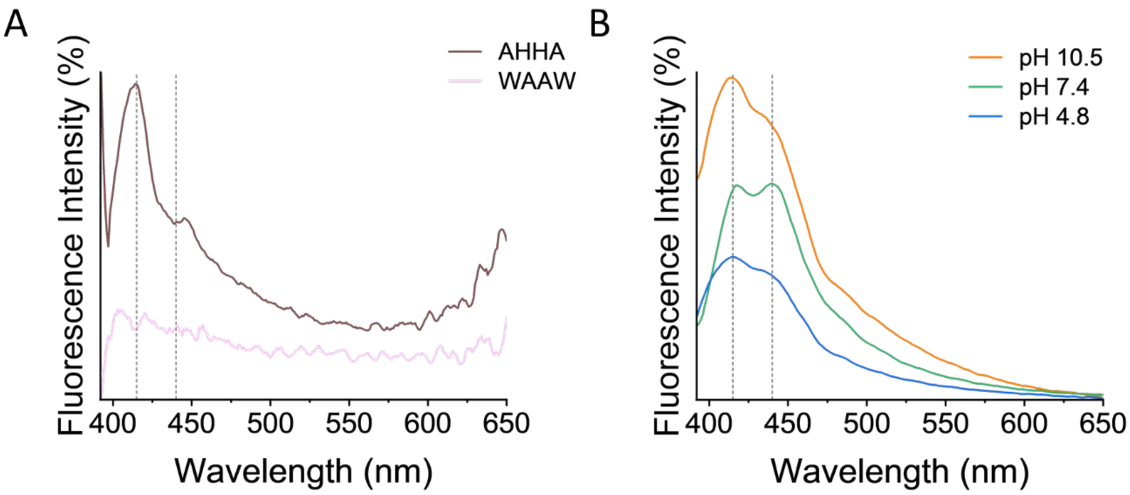

Figure S1. (A) A zoomed-in graph of the results presented in Figure 1C for the fluorescence intensity percentage of the control peptides AHHA and WAAW. (B) The fluorescence intensity percentage of pep1 (0.1 mM) in buffers (1 mM) at different pH values.

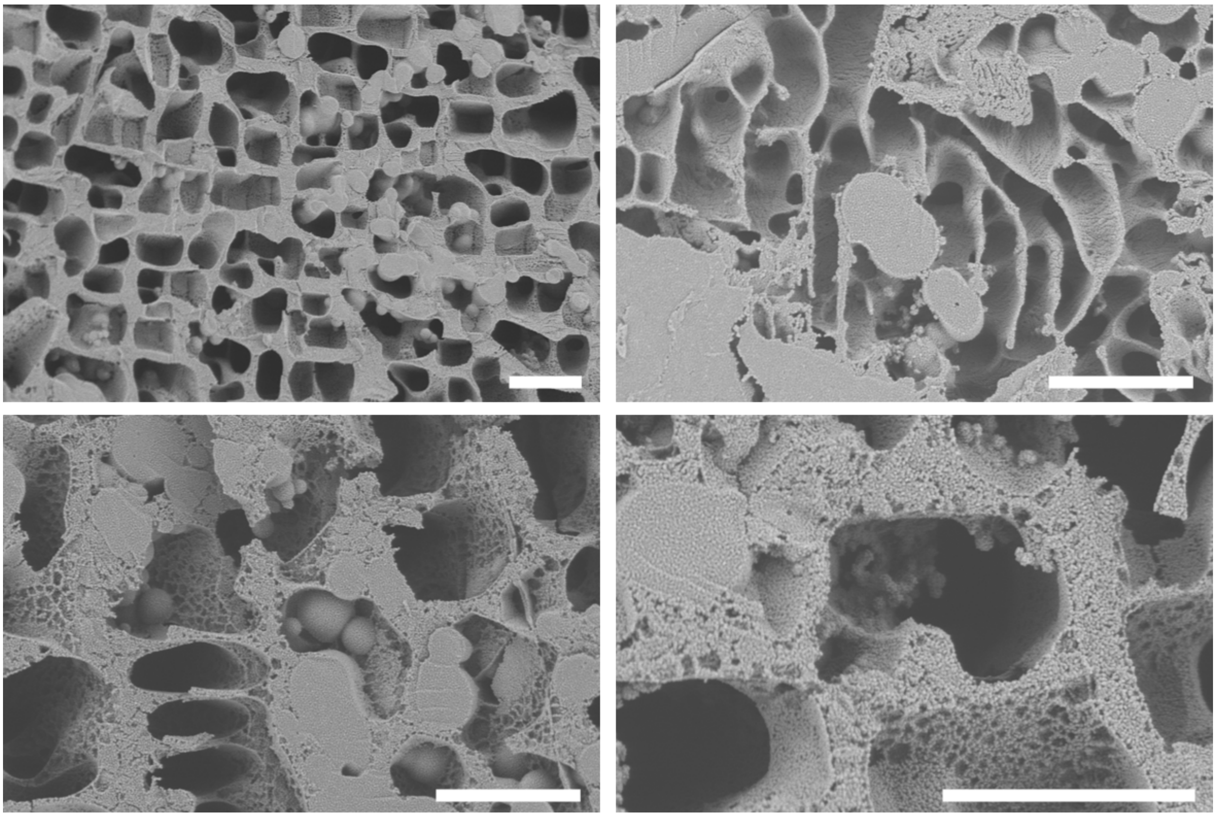

Figure S2. Cryo-SEM images of the coacervates of pep4 formed in solution (scale bars: 5 μm).

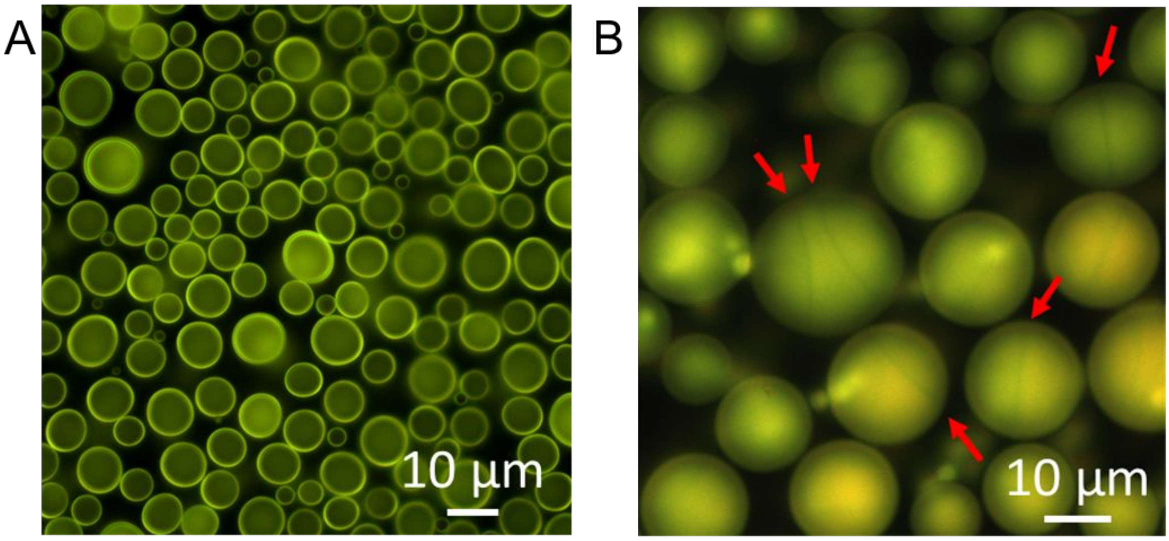

Figure S3. (A) A fluorescence microscopy image of coacervates of pep1 (10 mM) in phosphate buffer (pH 7.4, 25 mM, 0.2 M NaCl) formed during evaporation on a parafilm substrate. (B) A fluorescence microscopy image of coacervates of pep1 (8.3 mM) in phosphate buffer (pH 7.4, 25 mM, 0.4 M NaCl) with rhodamine 110 chloride (80 mg/L) formed during evaporation on a glass slide. Red arrows point to areas with low fluorescence that form during coalescence.

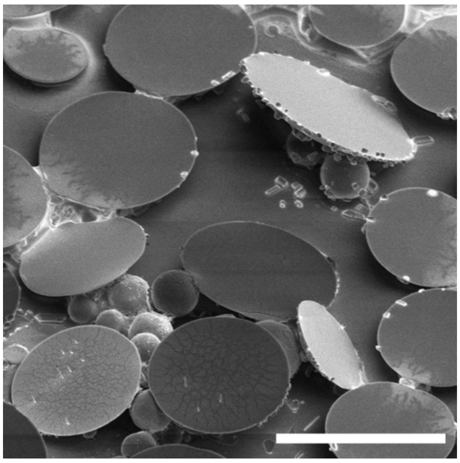

Figure S4. SEM image of coacervates of pep2 formed during evaporation (scale bar: 10 µm).

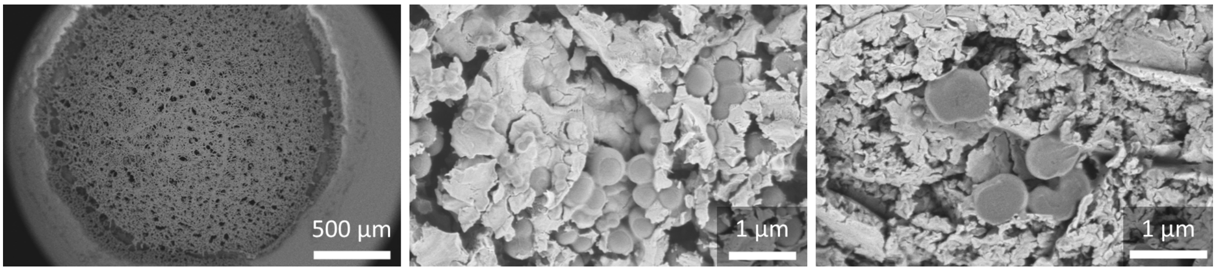

Figure S5. Cryo-SEM images of the evaporating droplet with coacervates of pep4 (left) and of the coacervates themselves (middle, right).

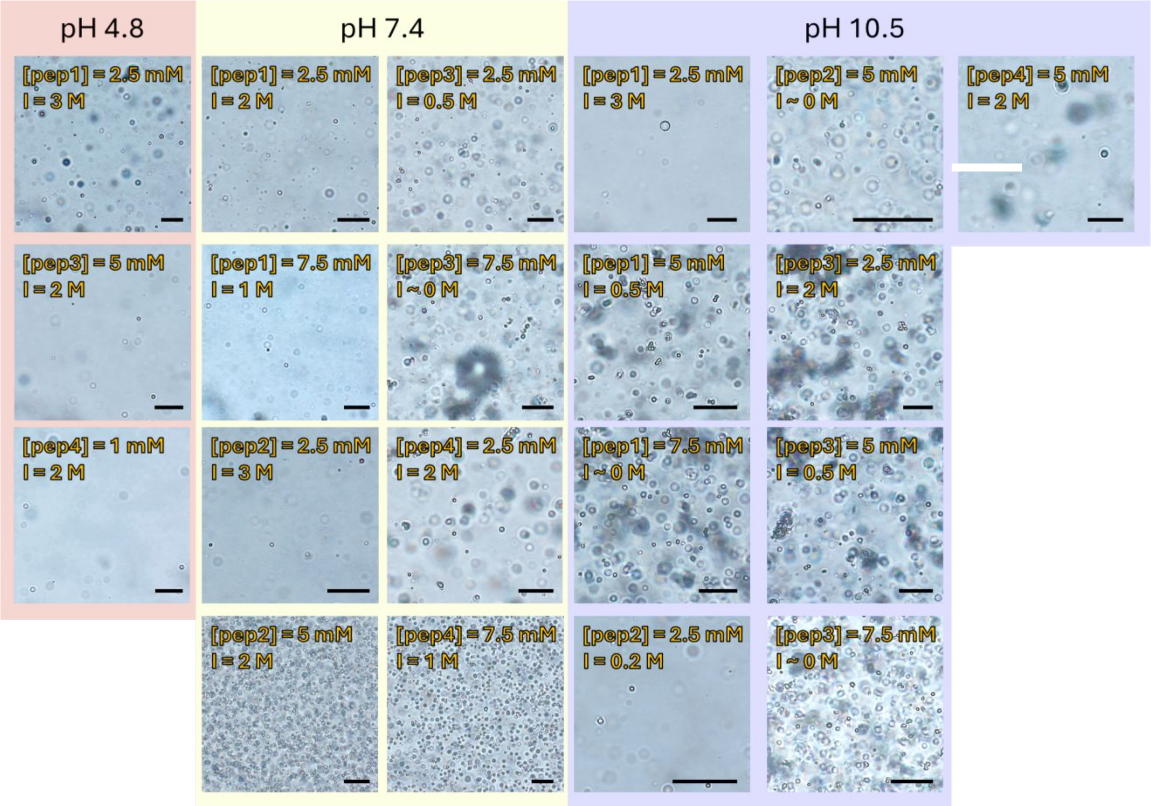

Figure S6. Transmitted light microscopy images of the coacervates formed at the lowest peptide concentration and ionic strength values that can produce coacervates in accordance with the phase diagram results shown in Fig. 4A (scale bars: 20 µm).

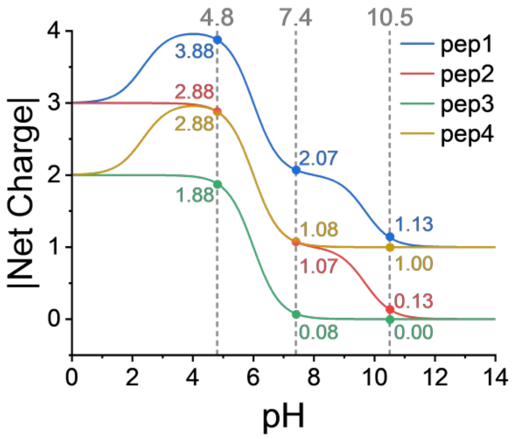

Figure S7. The absolute net charges of the four peptides versus the pH.

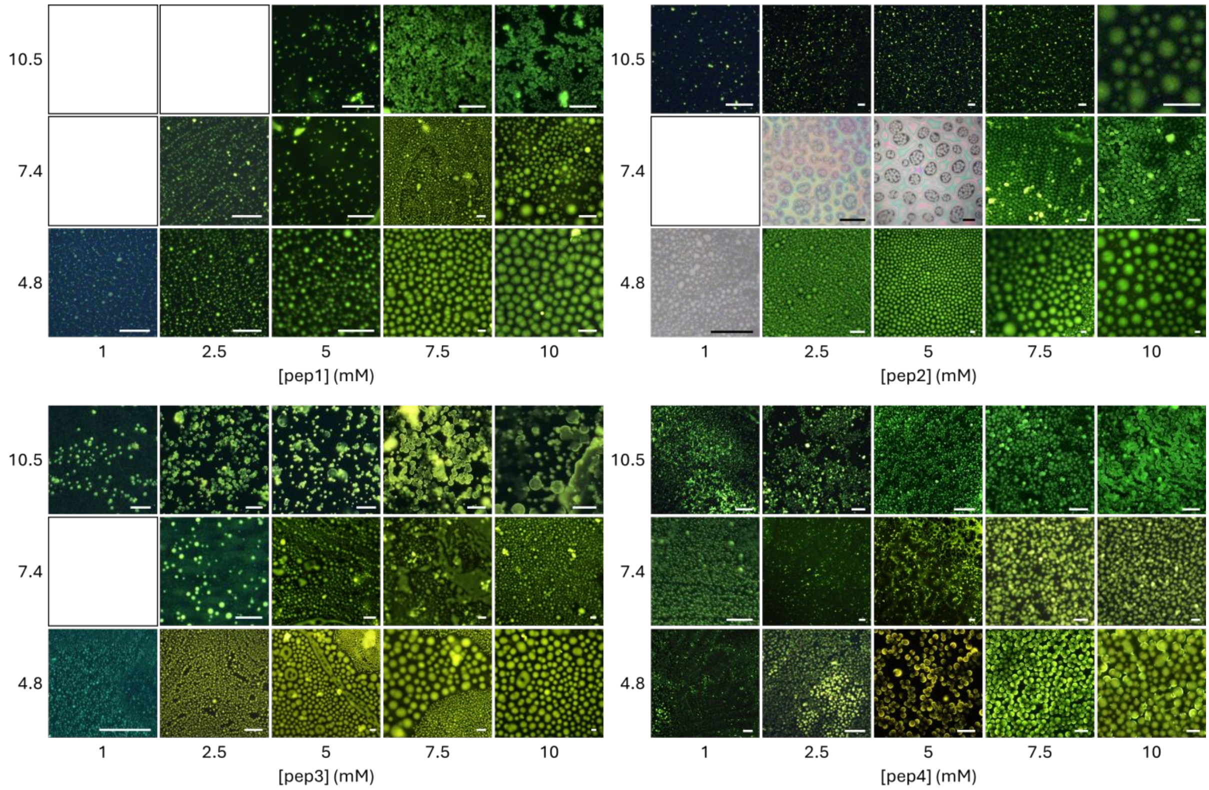

Figure S8. Fluorescence and transmitted light microscopy images of the coacervates formed during evaporation in accordance with the phase diagram results shown in Fig. 4B (scale bars: 20 μm). Transmitted light was used for the lower concentrations of pep2 due to its low fluorescence intensity.

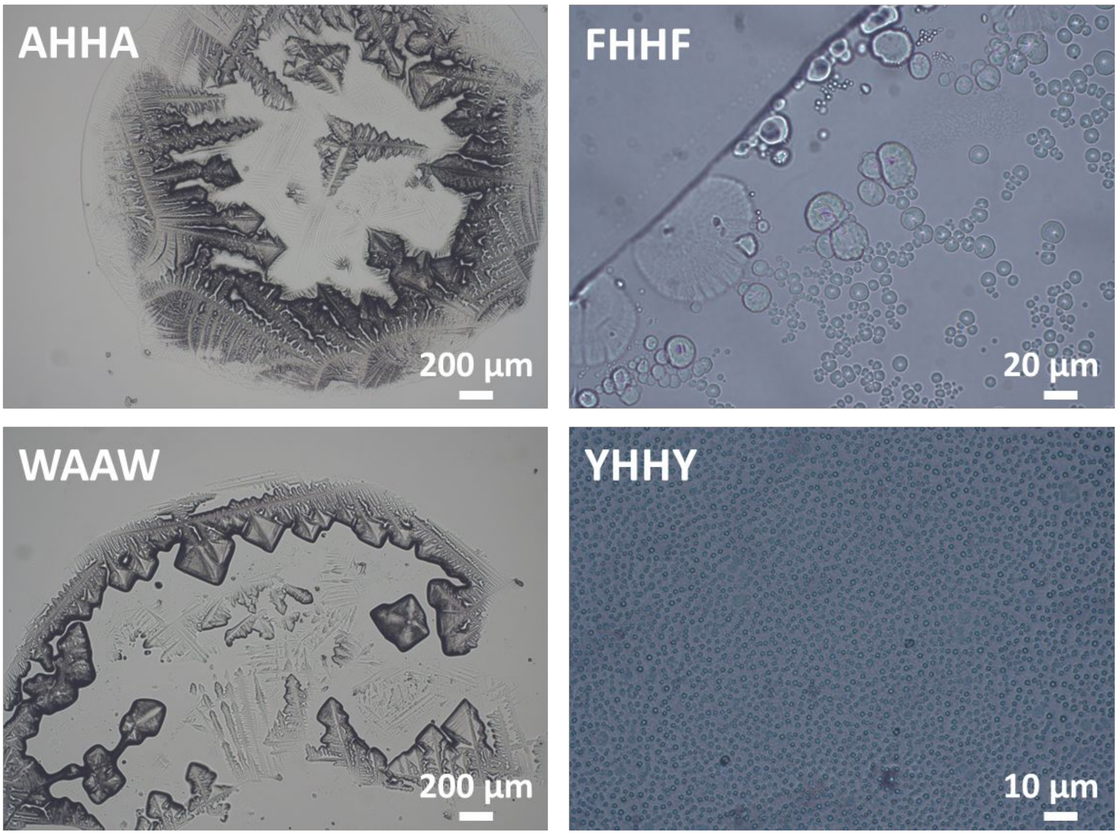

Figure S9. Transmitted light microscopy images showing the ability of YHHY and FHHF to form coacervates during evaporation in contrast to AHHA and WAAW.

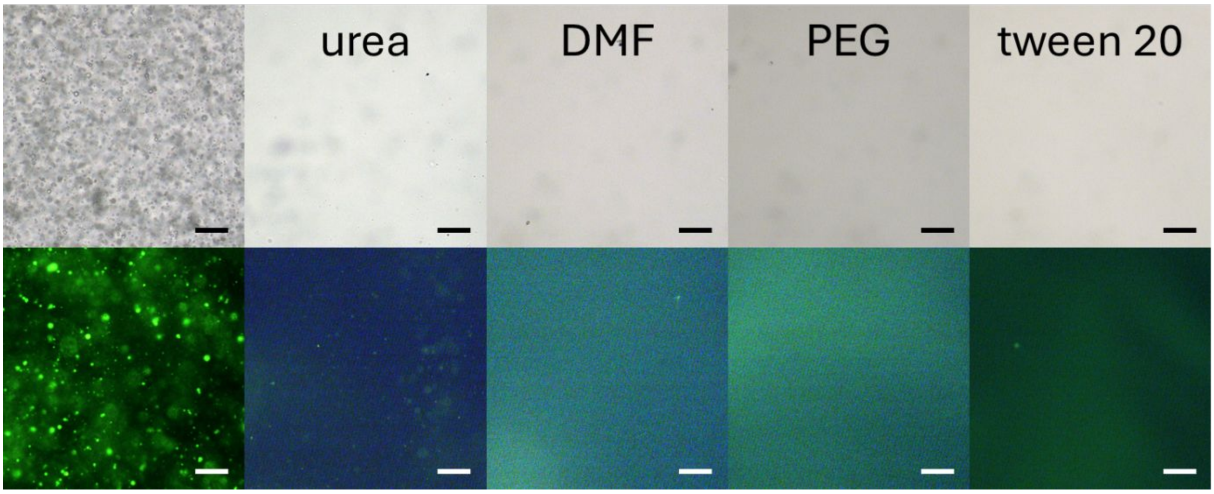

Figure S10. Transmitted light (top) and fluorescence (bottom) microscopy images showing the disassembly of the coacervates after addition of urea (0.7 M), or DMF, PEG, and tween 20 (10% v/v) (scale bars: 50 µm).

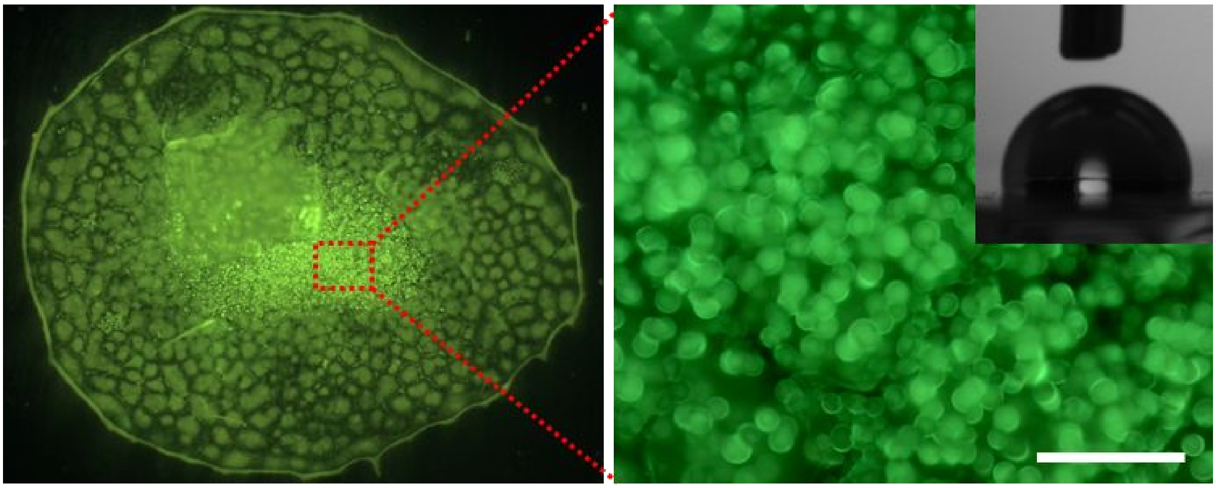

Figure S11. Fluorescence microscopy images of the deposition of coacervates of pep1 on a parafilm substrate under an external source of ethanol vapor. (scale bar: 50 μm). Inset: an image of the droplet under the vapor source.

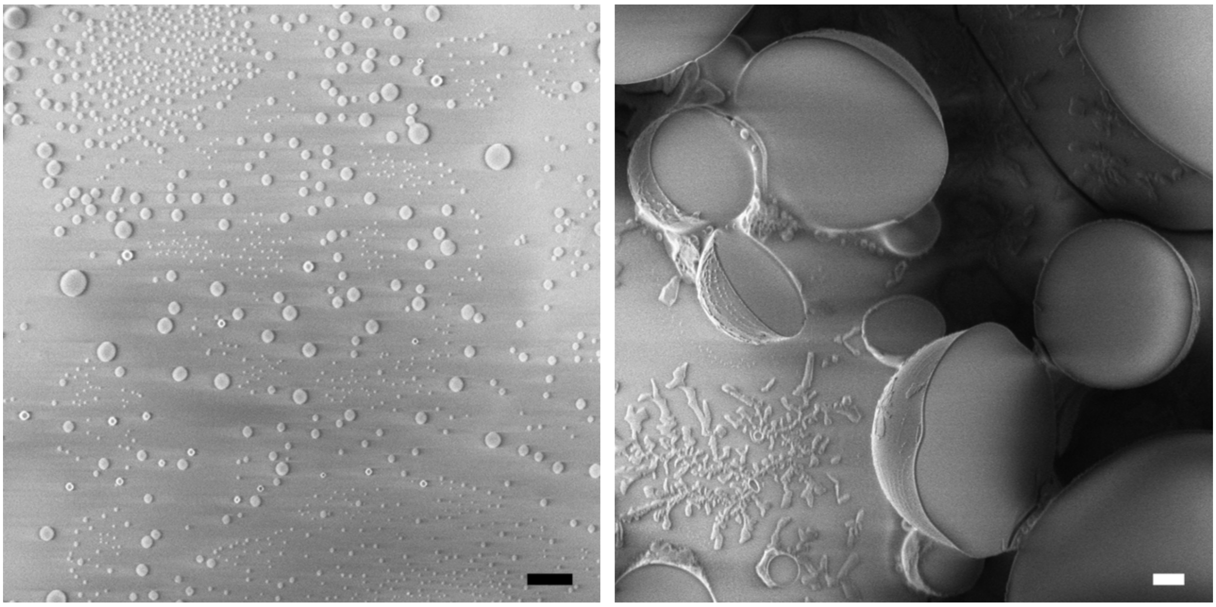

Figure S12. SEM images of coacervates of pep1 (10 mM) in phosphate buffer (pH 7.4, 25 mM, 0.3 M NaCl) formed during evaporation of droplets of 0.2 μL (left) and 5 μL (right) (scale bars: 1 μm).

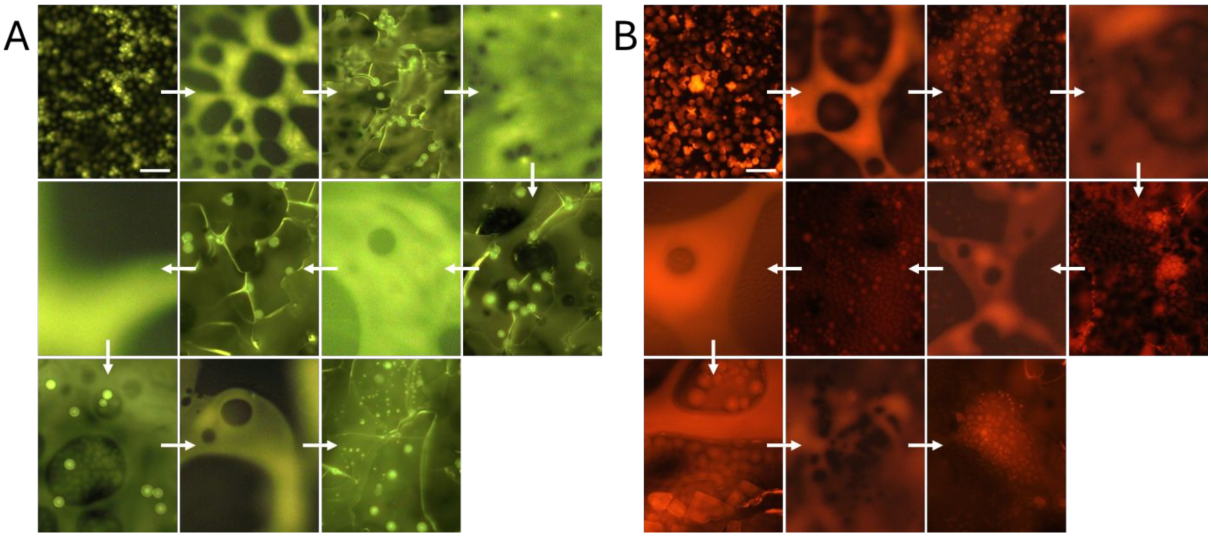

Figure S13. Fluorescence microscopy images of pep1 coacervates without (A) and with RhB (B) undergoing five cycles of RH increase and decrease, demonstrating their consistent ability to disassemble and reassemble with the changing humidity conditions (scale bars: 20 µm).

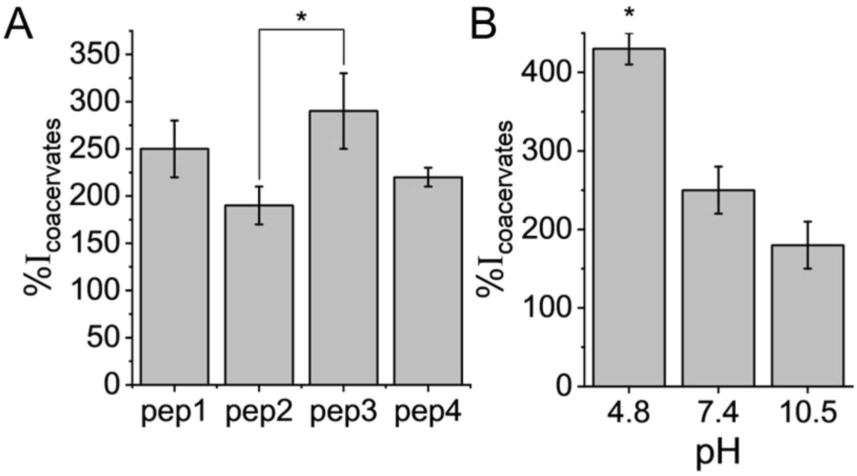

Figure S14. (A) The fluorescence of RhB (5 mg/L) inside the coacervates formed by the four peptides (5 mM, pH 7.4, 25 mM, 0.3 M NaCl). (B) The fluorescence of RhB (5 mg/L) inside coacervates of pep1 (5 mM) at different pH values (25 mM, 0.3 M NaCl). The data points and error bars represent the arithmetic mean and standard deviation values of triplicate measurements. The results were compared using one-way analysis of variance (ANOVA) test with Tukey-Kramer post hoc analysis. Statistically significant results were determined at $p < 0.05$ and marked with an asterisk.

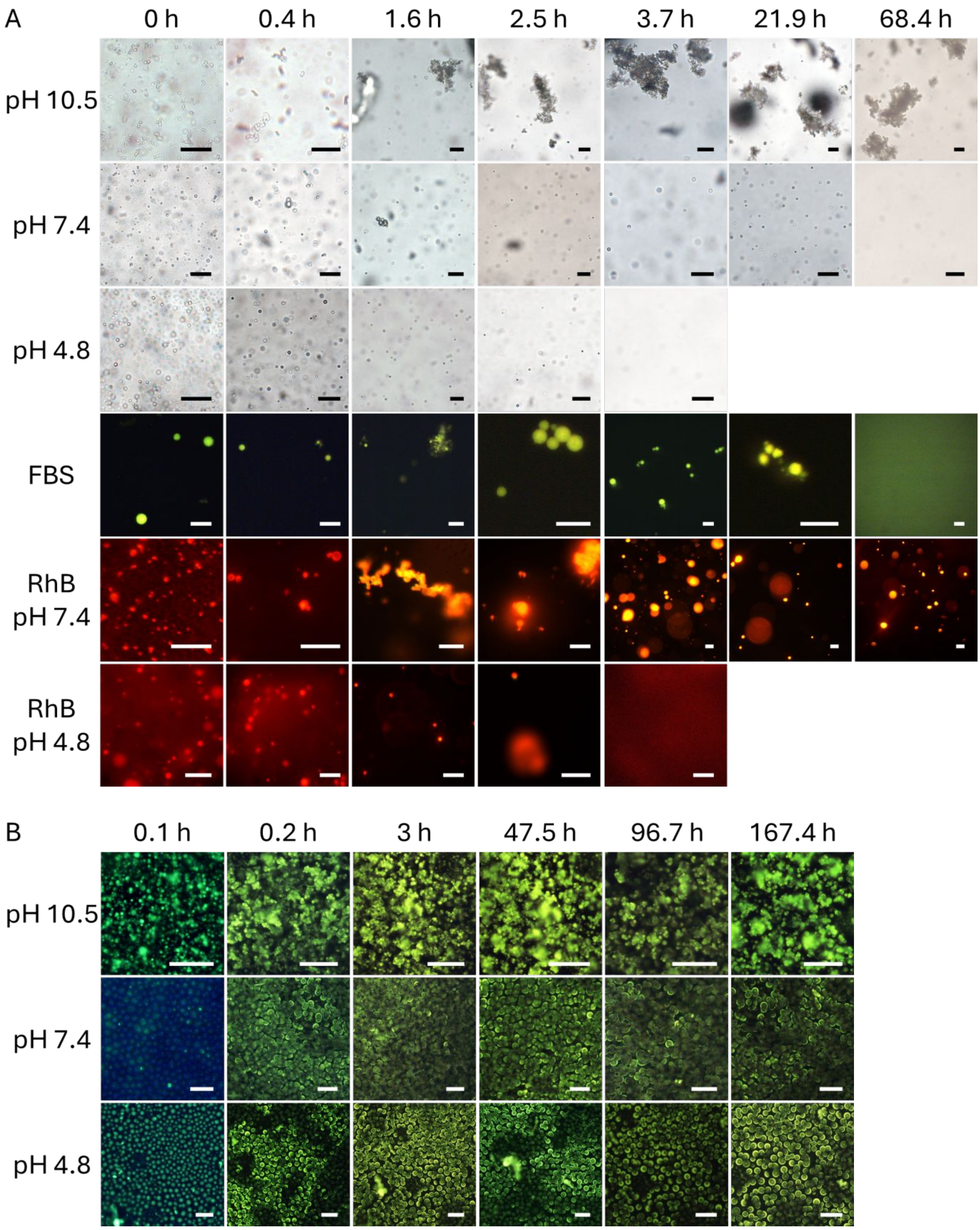

Figure S15. Transmitted light and fluorescence microscopy images showing the stability of the coacervates formed in solution (A) or during evaporation (B) at different conditions over time (scale bars: (A) 20 μm, (B) 50 μm).

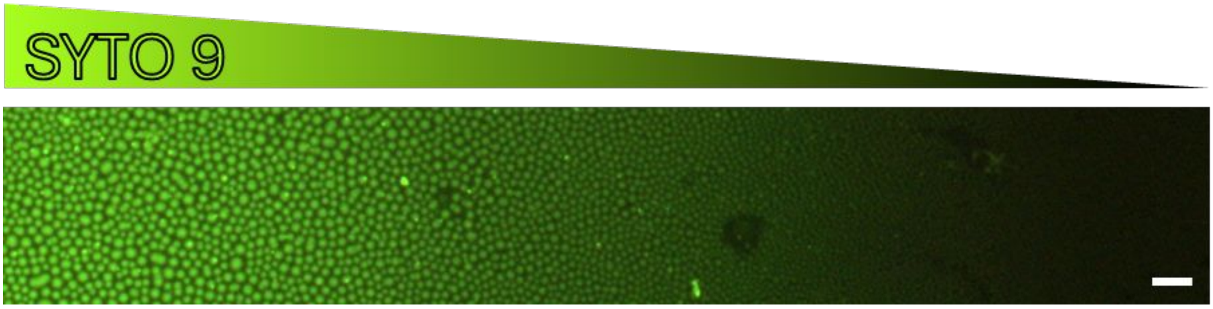

Figure S16. A fluorescence microscopy image of the concentration gradient of the fluorescent dye SYTO 9 inside the coacervates of pep 1 (scale bars: 50 µm).

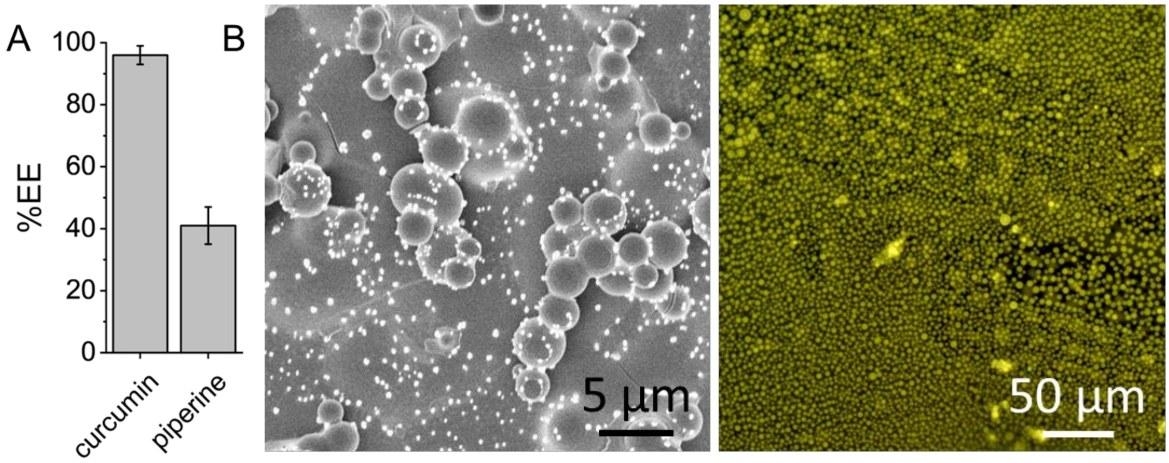

Figure S17. (A) Encapsulation efficiency values of curcumin and piperine in pep1 coacervates. (B) SEM (left) and fluorescence microscopy image (right) of coacervates of pep3 (5 mM) in acetate buffer (pH 4.8, 25 mM, 3 M NaCl) with curcumin (0.35 g/L) formed during evaporation.

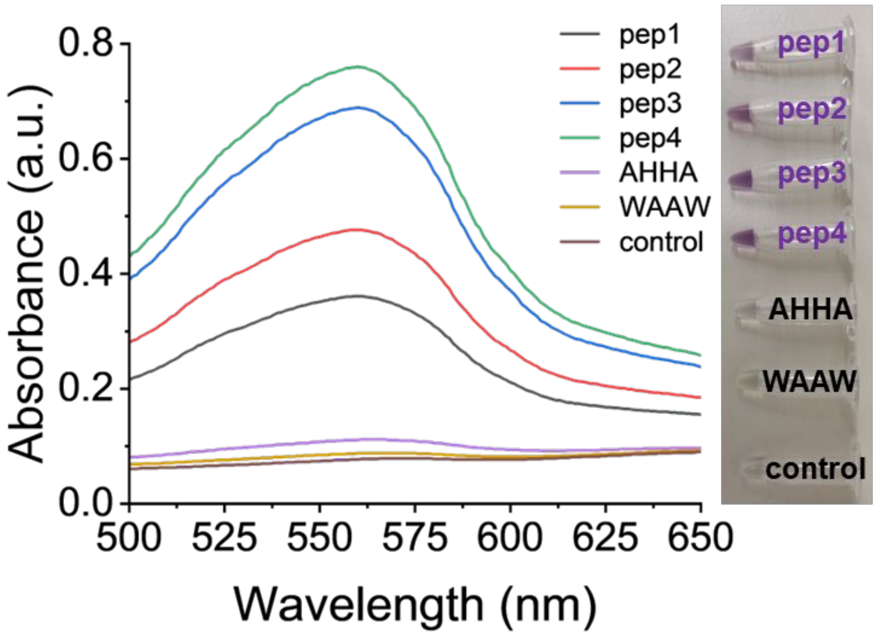

Figure S18. Absorbance spectra of different peptides mixed with $CuCl_2$ and with bicinchoninic acid. Inset: image depicting the color of the solutions.

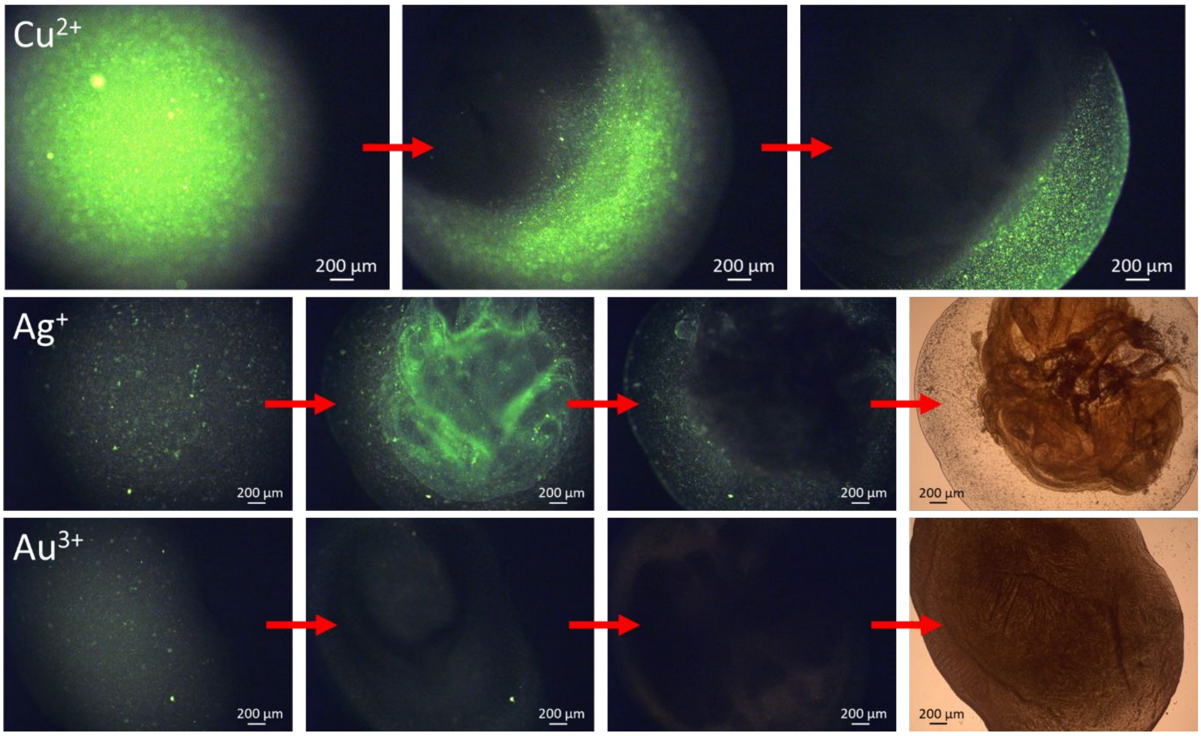

Figure S19. Fluorescence and transmitted light microscopy images of coacervates in solution of pep1 (10 mM) in carbonate buffer (pH 10.5, 25 mM, 3 M NaCl) with the addition of Cu^{2+} (9 mM), Ag^{+} (5 mM), or Au^{3+} ions (5 mM). The fluorescence images for each metal addition were taken in the same exposure parameters.

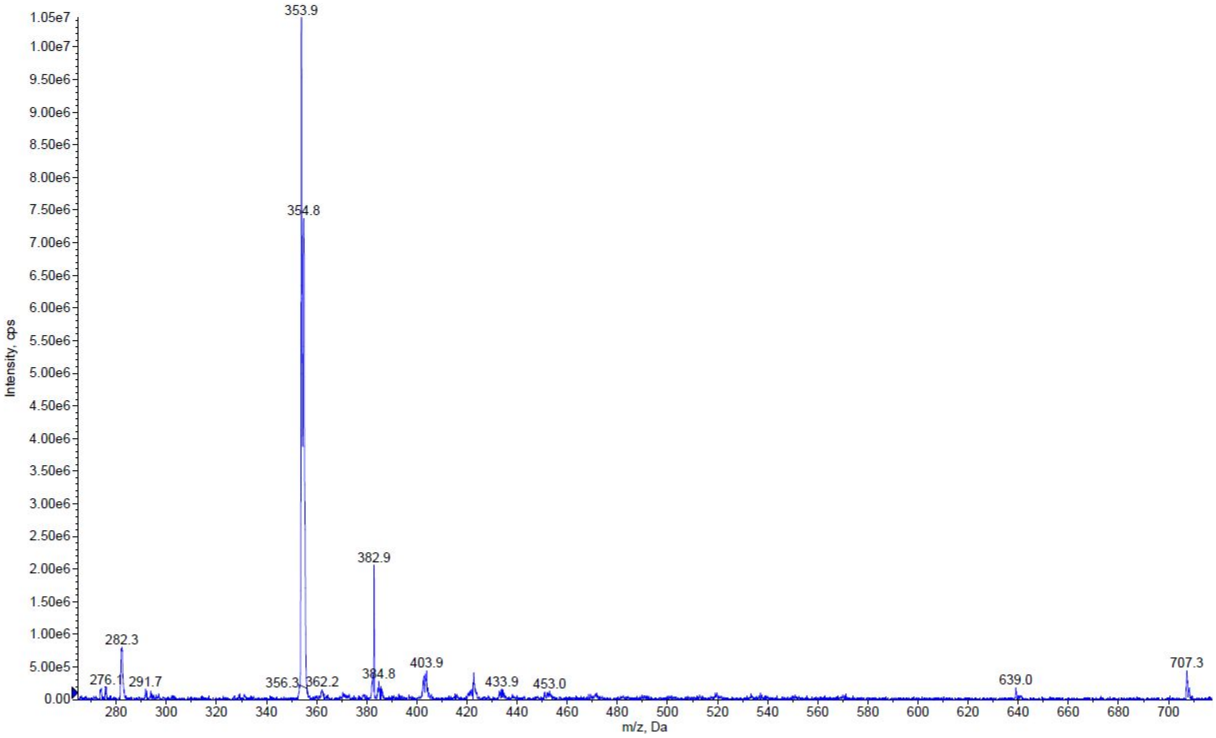

Figure S20. MS analysis of a mixture of pep4 (0.2 mM) in water with $CuCl_2$ (1 mM). The peaks at 353.9 and 354.8 Da correspond to the original peptide mass, while the peak at 382.9 Da corresponds with the mass of the oxidized peptide shown in Fig. 7G.

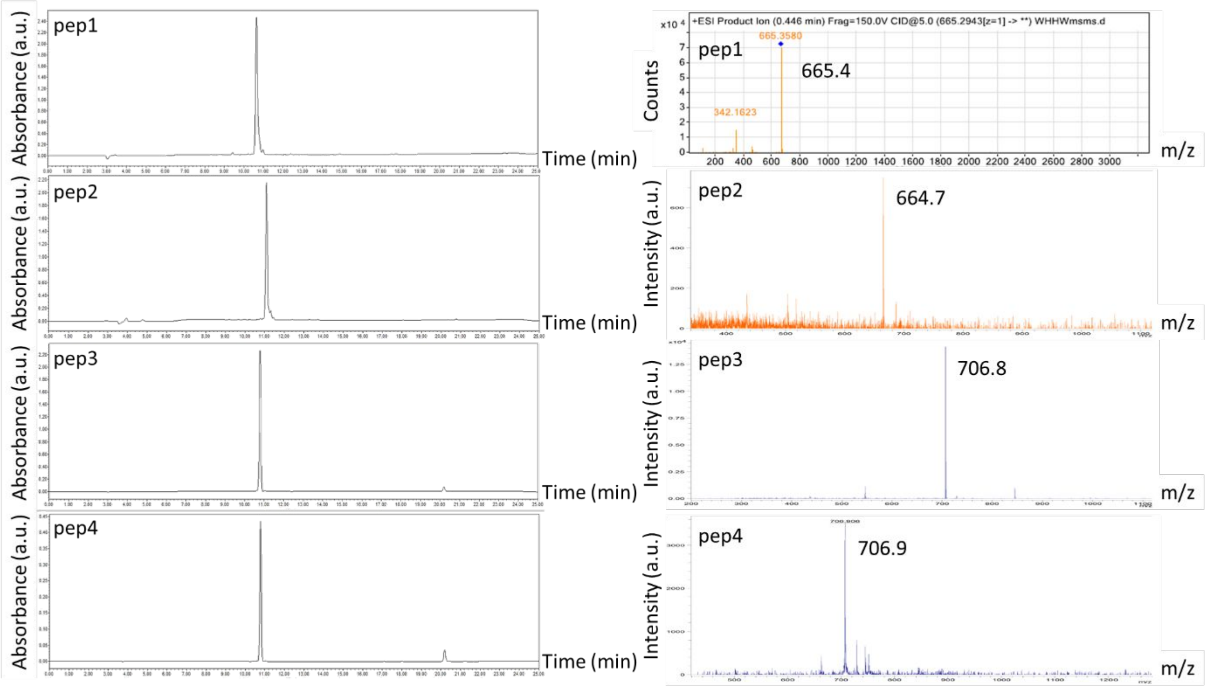

Figure S21. Analytical HPLC chromatograms (220 nm) and MS analyses for the four peptides.

References

(1) Zhang, Y.; Liu, G.; Ren, F.; Liu, N.; Tong, Y.; Li, Y.; Liu, A.; Wu, L.; Wang, P. Delivery of Curcumin Using Zein-Gum Arabic-Tannic Acid Composite Particles: Fabrication, Characterization, and in vitro Release Properties. *Front. nutr.* **2022**, *9*, 842850. DOI: 10.3389/fnut.2022.842850.

(2) Hua, C.; Yu, W.; Yang, M.; Cai, Q.; Gao, T.; Zhang, S.; Xu, H.; He, H.; Peng, N.; Liu, Y. Casein-pectin nanocomplexes as a potential oral delivery system for improving stability and bioactivity of curcumin. *Colloid. Polym. Sci.* **2021**, *299* (10), 1557-1566. DOI: 10.1007/s00396-021-04858-x.

(3) Mohammadian, M.; Salami, M.; Alavi, F.; Momen, S.; Emam-Djomeh, Z.; Moosavi-Movahedi, A. A. Fabrication and Characterization of Curcumin-Loaded Complex Coacervates Made of Gum Arabic and Whey Protein Nanofibrils. *Food Biophys.* **2019**, *14* (4), 425-436. DOI: 10.1007/s11483-019-09591-1.

(4) Liang, F.; Wang, M.; Hu, Y.; Guo, Z.; Yang, W. Efficient loading of curcumin into CTAB micelle-embedded silica particles for visualized pH detection. *Colloids Surf. Physicochem. Eng. Aspects* **2022**, *637*, 128250. DOI: https://doi.org/10.1016/j.colsurfa.2022.128250.

(5) De Leo, V.; Milano, F.; Mancini, E.; Comparelli, R.; Giotta, L.; Nacci, A.; Longobardi, F.; Garbetta, A.; Agostiano, A.; Catucci, L. Encapsulation of Curcumin-Loaded Liposomes for Colonic Drug Delivery in a pH-Responsive Polymer Cluster Using a pH-Driven and Organic Solvent-Free Process. In *Molecules*, 2018; Vol. 23.

(6) Coco, J. C.; Silvério, L. A.; Santos, É. M.; Sueiro, A. C.; Ataide, J. A.; Paiva-Santos, A. C.; Mazzola, P. G. Piperine Extraction and Encapsulation in Polycaprolactone Nanoparticles. In *Cosmetics*, 2023; Vol. 10.

(7) Alshehri, S.; Bukhari, S. I.; Imam, S. S.; Hussain, A.; Alghaith, A. F.; Altamimi, M. A.; AlAbdulkarim, A. S.; Almurshedi, A. Formulation of Piperine-Loaded Nanoemulsion: In Vitro Characterization, Ex Vivo Evaluation, and Cell Viability Assessment. *ACS Omega* **2023**, *8* (25), 22406-22413. DOI: 10.1021/acsomega.2c08187.

(8) Budama-Kilinc, Y. Piperine Nanoparticles for Topical Application: Preparation, Characterization, In vitro and In silico Evaluation. *ChemistrySelect* **2019**, *4* (40), 11693-11700. DOI: https://doi.org/10.1002/slct.201903266.
